# Stable Solid Electrolyte Interphase in Cylindrical Anode-Free Li-Metal NMC90 Batteries with Li_2_NiO_2_ Prelithiation and Fluorine-Rich Electrolytes for High Energy Density

**DOI:** 10.1021/acs.nanolett.5c01595

**Published:** 2025-05-22

**Authors:** Thitiphum Sangsanit, Ronnachai Songthan, Surat Prempluem, Worapol Tejangkura, Montree Sawangphruk

**Affiliations:** Centre of Excellence for Energy Storage Technology, Department of Chemical and Biomolecular Engineering, School of Energy Science and Engineering, 423058 Vidyasirimedhi Institute of Science and Technology, Rayong 21210, Thailand

**Keywords:** anode-free lithium-metal batteries, Li_2_NiO_2_ prelithiation, fluoroethylene carbonate, 18650 cylindrical cell, operando X-ray diffraction

## Abstract

This study advances anode-free lithium-metal batteries (AFLMBs) by integrating nickel-rich NMC90 cathodes and fluorine-rich electrolytes in large-format 18650 cylindrical cells. A key innovation is the incorporation of 10 wt % Li-rich Li_2_NiO_2_ as a prelithiation agent in the cathode, which mitigates initial lithium-loss and improves the Coulombic efficiency. The electrolyte includes 30% (v/v) fluoroethylene carbonate (FEC) as a cosolvent, which suppresses inactive lithium deposition and stabilizes the solid electrolyte interphase (SEI). Unlike conventional AFLMBs that require external pressure, this work uses a stainless-steel casing with a tailored jelly roll configuration to mechanically regulate lithium plating. The optimized cells deliver an energy density of 320 Wh/kg, maintain stable cycling over 140 cycles, and support 4C-rate operation. Post-mortem analysis reveals a LiF-rich SEI that extends the cycle life, while *operando* X-ray diffraction provides insights into structural evolution. This research offers a scalable strategy for high-energy AFLMBs through the synergy of prelithiation, electrolyte design, and mechanical stabilization.

## Graphical abstract

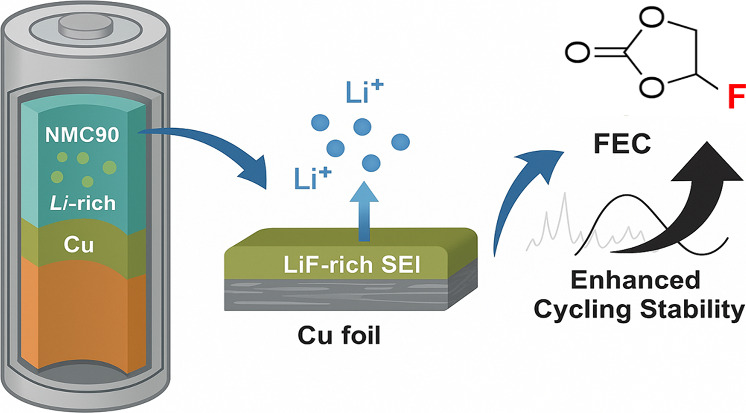

To surmount the challenges associated with extending the driving range of electric vehicles beyond 1000 km per single charge, the adoption of anode-free or metal reservoir-free battery configurations is increasingly recognized as an essential strategy, primarily attributed to their inherently high energy density resultant from the omission of anode active materials.^1–3^ Nevertheless, the pathway to commercial deployment of such technologies is obstructed by a multitude of technical hurdles. Within the ambit of a full cell configuration, the constraint posed by the finite lithium reservoir necessitates a nuanced approach to counteract the critical issue of initial lithium depletion observed on the bare copper (Cu) current collectors during the lithium plating and stripping processes, as opposed to intercalation into an anode material.^4^ The technique of prelithiation is posited as a viable remedy;^5^ however, traditional prelithiation agents, characterized by their lithium-metal composition,^6,7^ suffer from air sensitivity and a lack of compatibility with standard battery production protocols, which are predominantly executed within dry-room conditions. To navigate these challenges, our research introduces lithium nickel oxide (Li_2_NiO_2_)^8^ as a prelithiation agent, offering an effective means to compensate for the initial lithium loss encountered. Modification of Cu foil with a thin lithiophilic layer (e.g., graphene oxide^9^ or Al_2_O_3_ with an ionic liquid^10^) can also be applied. Additionally, in the context of extended cycling durability, the modulation of pressure exerted upon the Cu foil in anode-free pouch cells is deemed pivotal for the uniform deposition of lithium.^11^ The use of a cylindrical casing, while imposing limitations on the adjustment of the external pressure, presents an opportunity to explore the incorporation of a tailored jelly roll configuration within the casing to impose beneficial pressure constraints, thereby enhancing the cell performance. Furthermore, the composition of the electrolyte emerges as a critical factor in curtailing the growth of lithium dendrites, as reported in the literature. The use of dual-salt fluoride cosolvents (TTE/FEC),^12^ as well as high salt concentrations (e.g., 3 M LiFSI^13,14^ ), can also contribute to the formation of a stable SEI and extended cycle life. In terms of fluorinated solvents, 30% (v/v) FEC^15^ was employed. While significantly high salt concentrations (3 M) are generally more suitable for anode-free systems, they may not be ideal for cylindrical cells^16^ due to practical limitations including increased viscosity, poor wettability,^17^ and Li^+^ diffusivity^18^ as well as higher cost. Therefore, a moderately high concentration of 1.2 M LiPF_6_ was used in this study. Besides, the discourse within the existing literature predominantly revolves around small-scale experimental setups, such as coin cells, which do not adequately mirror the complexities and operational parameters of full-scale lithium battery configurations, including cylindrical, pouch, or prismatic cells,^19^ as reviewed in Table S1.

This study examines the production and characterization of anode-free 18650 cells, standardized by a battery checklist (Table S2), and utilizes a nickel-rich NMC90 cathode to amplify energy density, as illustrated in Figures 1a–c, S1 (18650 cell pilot-scale production), and S2 (characterization and Rietveld refinement of NMC90) and Table S3 (wavelength-dispersive X-ray fluorescence characterization of the NMC90). These cells were fabricated by employing a pilot-scale 18650-cell production process.^20^ The anode is exclusively composed of bare Cu foil (Figure 1a), while the separator incorporates an Al_2_O_3_-coated PP/PE/PP to diminish the risk of internal short circuits (Figure 1d). The electrolyte formulation, developed in-house, comprises 1.2 M LiPF_6_ in a mixture of EC/DEC/EMC/FEC.^21^ Upon electrolyte infusion and subsequent cell assembly (Figure 1e), the cells underwent evaluation for their electrochemical performance and potential application viability, including deployment in drone technologies (Figure 1f) for which cylindrical cells have been rarely used because they are rather heavier compared to the LiPo lithium-ion batteries. This research aims to elucidate the feasibility of commercializing anode-free 18650 cells by bridging the gap between electrochemical efficacy and practical engineering considerations.

**Figure 1:**
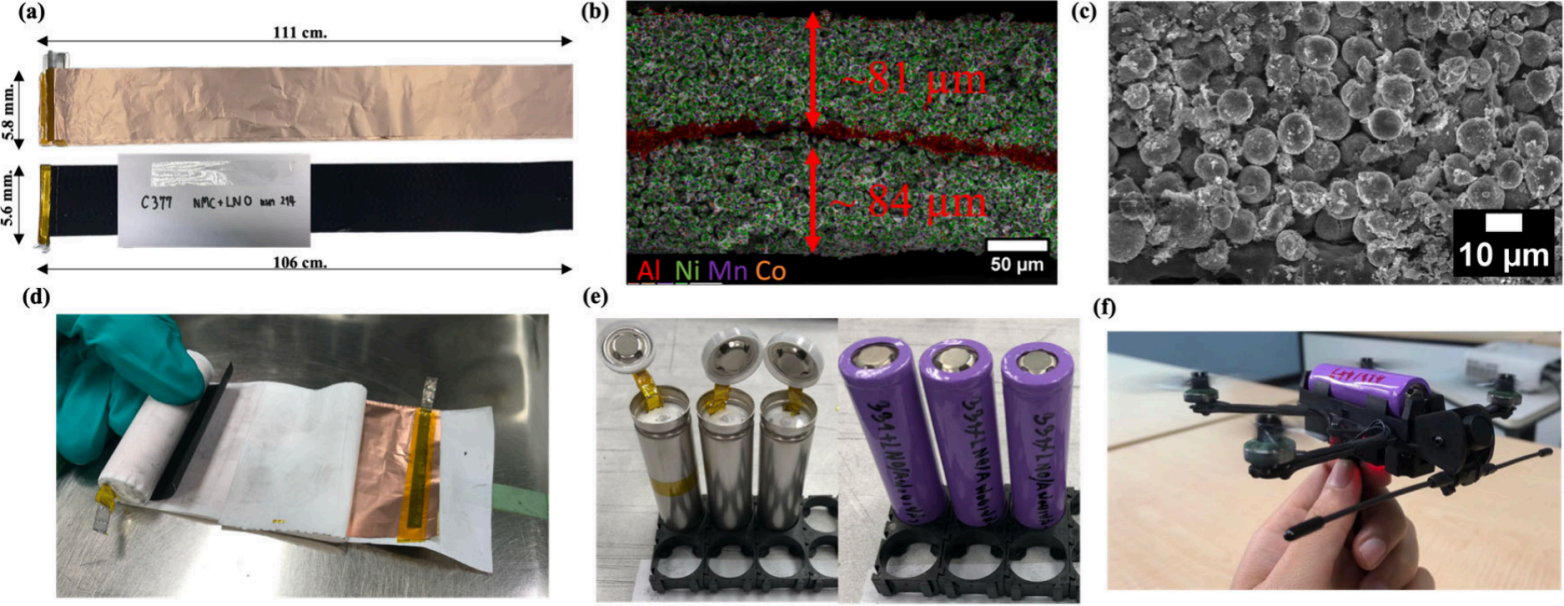
Fabrication and evaluation of anode-free 18650 cells. (a) Cu foil utilized as the anode substrate alongside the cathode composed of NMC90 with an additional 10 wt % Li_2_NiO_2_. (b and c) SEM images illustrating the cathode’s microstructural thickness. (d) Assembled anode-free 18650 jelly roll, showcasing its coiled structure. (e) Anode-free cells captured both prior to and subsequent to the infusion of electrolyte and the completion of cell assembly. (f) Commercial drone powered by the anode-free 18650 cell, demonstrating the practical application of the cell in real-world scenarios replacing the conventional LiPo cells.

The formation cycle of lithium-metal batteries catalyzes the genesis of a solid electrolyte interphase (SEI)^22^ on a graphite anode, a critical process that invariably restricts the initial Coulombic efficiency (ICE) to below unity. This limitation is notably exacerbated in anode-free battery configurations, wherein the nonlithiophilic characteristics of bare Cu foil^23^ precipitate voltage overshoots and substantial initial losses attributable to SEI formation and the accrual of inactive lithium, deviating markedly from traditional lithiation mechanisms. In response to this, our investigation incorporates Li-rich Li_2_NiO_2_ as a novel prelithiation agent. Contrary to the layered structure typified by the *R*3*m* space group of LiNiO_2_ cathodes, Li_2_NiO_2_ is distinguished by an orthorhombic structure within the *Immm* space group, facilitating lithium integration not only within a two-dimensional plane but also along the *Z* axis, albeit leading to structural collapse of the positional charge, as shown through characterization and Rietveld refinement in Figure S3. Despite this, Li_2_NiO_2_ is documented to irreversibly deliver an energy capacity of 340 mAh/g, corroborated by both of our findings (Figure S4: electrochemical behavior of Li_2_NiO_2_ 18650 cells) and extant literature,^8^ within a voltage window identical with that of NMC90 cathodes. Due to its comparable chemical properties, Li_2_NiO_2_ seamlessly integrates into the cathode slurry alongside NMC90, binder, and conductive carbon. Consequently, we augmented the NMC90 cathode with a 10% weight proportion of Li_2_NiO_2_, serving as a prelithiation agent, with detailed specifications provided in Table S4. Our experimental data reveal that the anode-free NMC90 cells, infused with 10% Li_2_NiO_2_, exhibit an ICE of 82.88 ± 0.72% (*n* = 7), as shown in Figure 2a. This performance substantially surpasses that of cells devoid of Li_2_NiO_2_ (Figure S5: initial cycle of anode-free NMC90 without Li_2_NiO_2_), albeit still trailing behind conventional graphite/NMC90 cells (Figure S6: initial cycle of graphite/NMC90). X-ray diffraction (XRD) analysis preformation identified a pronounced Li_2_NiO_2_ peak, constituting 11.9% by weight as per Rietveld refinement, which notably vanished postformation, as discussed again in the post-mortem part, implicating the irreversible lithium contribution from Li_2_NiO_2_ toward mitigating initial losses. This underscores the imperative to moderate Li_2_NiO_2_ concentrations to preserve the reversible capacity. Subsequent to their assembly, the cells were subjected to capacity evaluation at a C/10 rate, yielding a discharge capacity of 3407.30 ± 115.15 mAh (*n* = 5), as shown in Figure 2a,b. This translates to a specific capacity of 189.29 mAh/g based on the active mass and 210.32 mAh/g for the NMC90 component. Additionally, the exclusion of graphite from the cell composition resulted in an elevation of the nominal voltage to 3.8 V, surpassing the 3.6 V typically observed in conventional graphite/NMC cells, as delineated in Figure S6. These metrics underscore the potential of the cell to deliver energy densities of 320 Wh/kg at the cell level and 510 Wh/kg at the jelly roll level, as catalogued in Table S4.

**Figure 2:**
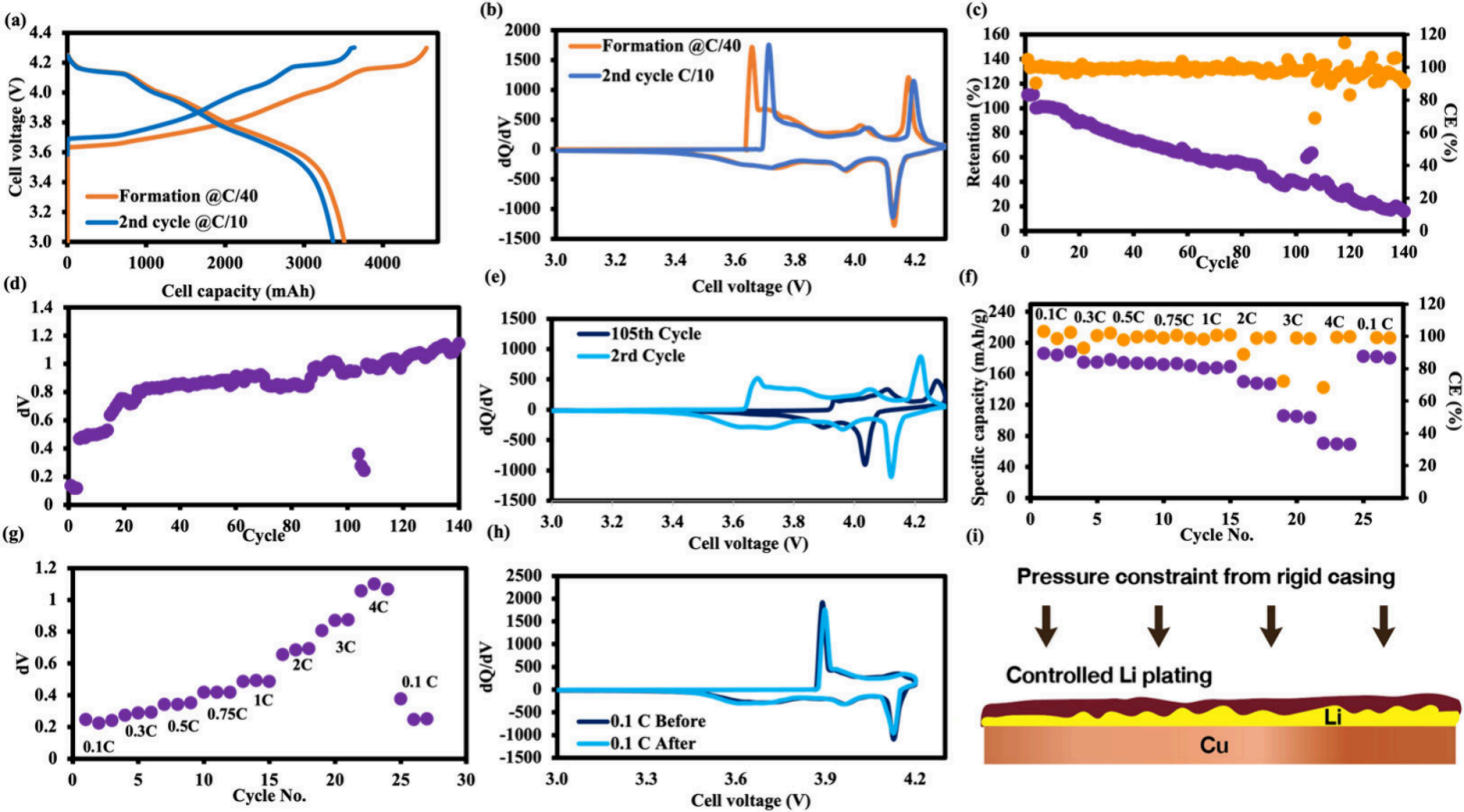
Electrochemical behavior of 18650 anode-free cells: (a) charge–discharge profile, (b) d*Q*/d*V* plot profile during formation cycle at C/40 and capacity determination at C/10. Stability tests: (c) capacity retention (%); (d) different voltage (d*V*); (e) d*Q*/d*V* plot at 0.5 CCCV charge and 1 CC. Rate performance: (f) specific capacity; (g) different voltage; (h) d*Q*/d*V* plot. (i) Schematic picture describing the benefits of the rigid casing for Li plating–stripping.

In the context of electrochemical stability, the anode-free 18650-format cell utilizing NMC90 cathodes augmented with 10 wt % Li_2_NiO_2_ demonstrated operational durability for up to 140 cycles, with the CE approaching 100% during early-to-mid cycling (Figure 2c,d). However, beginning after approximately 100 cycles, the CE exhibited sharp fluctuations above and below 100%, concurrent with a drop in the reversible capacity to below 40%. This behavior can be attributed to the constant discharge current (1C), which was calculated based on the initial capacity; as capacity diminished, the effective C rate increased to an estimated 2C–4C. At these elevated rates, the SEI is destabilized, particularly the LiF-rich components, as confirmed by X-ray photoelectron spectroscopy (XPS; Figure S10c) and post-mortem analysis (Figure 3c). SEI degradation may temporarily release trapped lithium species into the electrolyte, leading to a transient reintercalation into the cathode and a spike in the CE above 100%. However, this phenomenon is followed by irreversible lithium consumption as fresh lithium or exposed Cu surfaces react with the electrolyte, ultimately accelerating cell degradation. This is clearly reflected in the electrochemical data in Figure S7 (electrochemical behavior of the 18650 NMC90 plus 10 wt % Li_2_NiO_2_ anode-free cell after 100 cycles). Differential capacity (d*Q*/d*V*) analysis further supports this interpretation, showing a progressive loss of characteristic redox peaks beyond 105 cycles (Figure 2e), indicative of lithium inventory depletion and cathode degradation. These findings are consistent with previous reports in anode-free systems, where high-rate cycling and lithium limitation contribute to nonequilibrium CE behavior and capacity fade. Besides, we benchmarked our anode-free cell against in-house graphite//NMC90 and commercial LG 18650HG2 graphite//NMC cells (Figure S8). After 100 cycles, the anode-free cell showed an average CE that was lower by 0.55–0.62%, and a higher standard deviation than those of both graphite-based systems. This result highlights the inherently less stable and more variable nature of the lithium plating/stripping mechanism, as opposed to the well-established and reversible intercalation process in graphite anodes.^24^ In practical terms, the anode-free cell fulfilled the 80% capacity retention benchmark—commonly referenced for EV battery replacement, for 30 cycles. Moreover, it maintained over 2000 mAh/cell for 65 cycles, comparable to the nominal capacity of commercial graphite∥NMC 18650 cells^25^ and exceeded 1200 mAh/cell for 115 cycles, aligning with commercial graphite∥LFP 18650 cells^26^ (Figure S9). While the cycle life remains shorter than that in mature Li-ion chemistries, the successful demonstration of high-energy-density, anode-free operation in a commercial cylindrical format represents a substantial advancement beyond previous studies, which have largely focused on coin or pouch cells under idealized conditions. Additionally, prior anode-free studies have typically relied on external mechanical pressure to stabilize lithium deposition and suppress SEI degradation.^4,27^ In contrast, our design leverages the mechanical confinement of a stainless-steel cylindrical casing and a tailored jelly roll structure, demonstrating a performance under more realistic, scalable engineering constraints.

**Figure 3:**
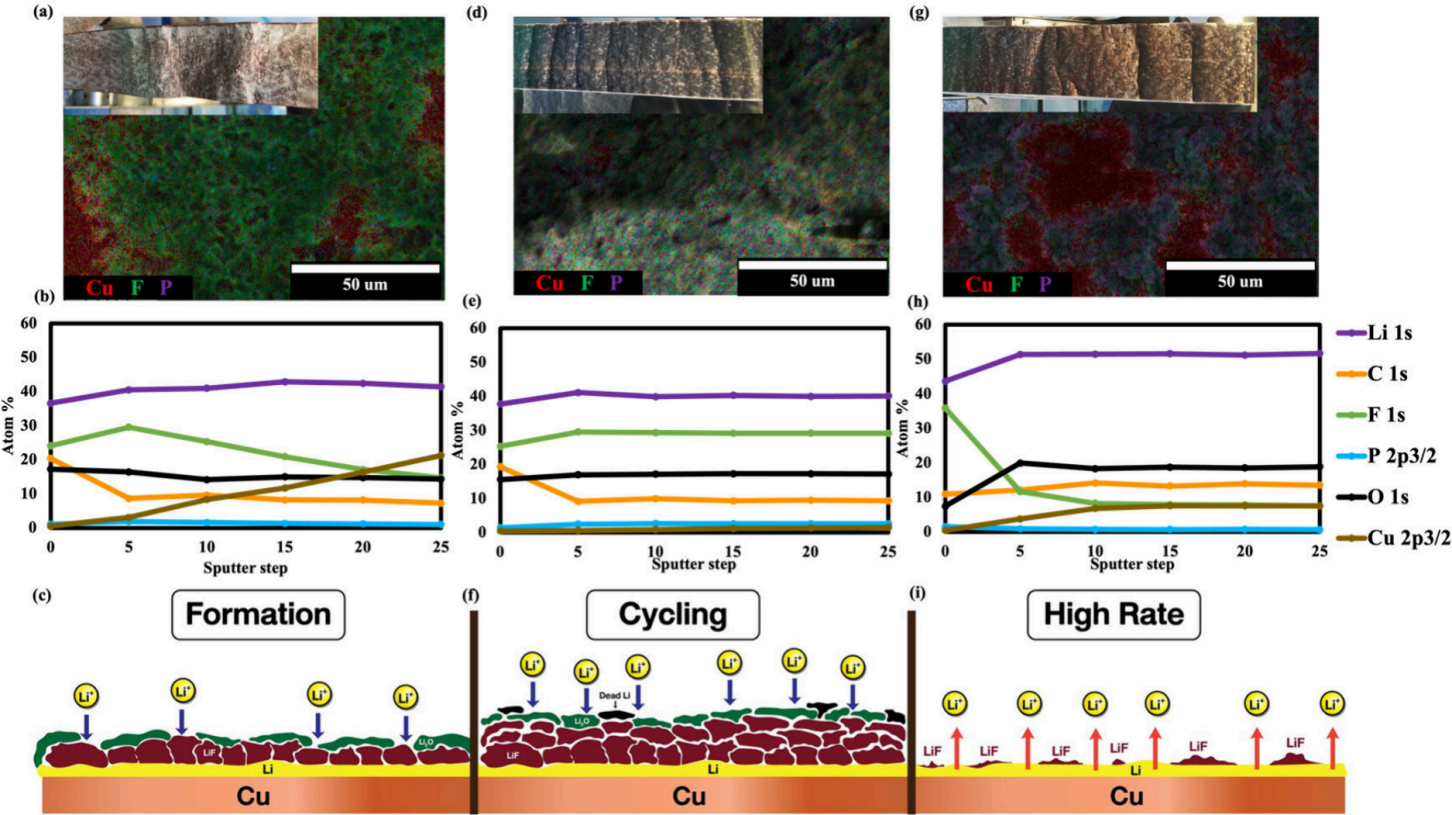
Post-mortem analysis of anode-free cells: (a–c) formation cycle; (d–f) stability test; (g–i) rate performance test. (a–g) SEM images and EDS analysis (noted in the small pictures at the top images showing photographs of the electrodes after disassembly in an Ar-filled glovebox). (b, e, and h) XPS elemental analysis at the surface and different depths. (c, f, and i) Schematic diagram describing Li plating and stripping.

In this vein, we conceived a comparative experiment that entailed designing a jelly roll with a diameter marginally less than its enclosing casing, thereby generating a mismatch in fit. Within this identical electrode configuration, the mismatched cells exhibited a capacity for up to 60 cycles of operation (refer to Figure S10b,c: cycling performance of the unfiltered jelly roll). Contrastingly, the utilization of a rigid 18650 casing appeared to augment the cycling stability attributable to the jelly roll’s expansion during charge cycles, thus preserving the cell’s volumetric constancy.

Concerning the rate capability, the anode-free NMC90 with a 10 wt % Li_2_NiO_2_ configuration was capable of sustaining operations at up to a 4C rate (Figure 2f,g) without noticeable deterioration in the d*Q*/d*V* peaks (Figure 2h), starkly contrasting with cells comprising mismatched jelly rolls. These latter cells could only achieve up to a 3C rate and were unable to recover full capacity at reduced rates, as documented in Figure S10 (high-rate performance of the unfiltered jelly rolls). This phenomenon ostensibly contributes to modulation of the pressure dynamics, influencing the plating and stripping mechanisms, as depicted in Figure 2i. In a controlled experiment within a glass cell under an argon atmosphere, anode-free jelly rolls were analyzed to elucidate the plating process. The findings, illustrated in Figure S11 and the accompanying schematic, underscore the critical role of physical constraints in mitigating continuous lithium plating on the Cu foil and instead promoting lithium-metal growth at the jelly roll’s edges during charging phases.

Post-mortem analysis was necessary for a deep investigation. The anode-free cells were discharged to 3.0 V and disassembled in an Ar-filled environment. XRD was used to characterize the NMC90 major phase and the Li_2_NiO_2_ minor phase (Figure S12: XRD Rietveld refinement of the NMC90 + 10 wt % Li_2_NiO_2_ electrode). As mentioned in the formation section, the minor phase was observed only in the fresh electrode, with 2Li occupancy, indicating a Li-rich material. After the formation cycle, this phase disappeared. No clear crystalline phases emerged, presuming that it transformed into an amorphous state and remained on the surface. For the main NMC90 phase, even after discharging, the C lattice of the sample after formation, cycling, and high-rate tests expanded, indicating lower lithium occupancy in the structure, especially after cycling, suggesting Li^+^ loss from the cathode.

White-gray species accumulate on the Cu foil for the anode side after formation (see an inset picture in Figure 3a). Therefore, XPS and scanning electron microscopy (SEM)–energy-dispersive spectroscopy (EDS) mapping were performed to analyze the species of this growth layer. As shown in Figure 3b, elemental analysis of XPS indicated that the main species on the Cu surface was Li, and the content of Li species remained throughout the 200 nm depth profile under Ar bombardment. F species was the main observed species, mainly belonging to the SEI formed by a FEC cosolvent. Conversely, the signal of Cu species was clearer following the trend of sputtering time and clearly observed around 120–160 nm, indicating the depth of SEI and lithium species on the surface of the Cu foil.

For the species fitting (Figure S13a: C 1s, Li 1s, and O 1s XPS narrow scans and fitting after the formation cycle), a thin layer of Li metal was observed on the surface, indicating the presence of dead lithium or residual lithium. However, the main species that persisted through 200 nm was LiF, originating from a FEC cosolvent, and Li–O, indicating Li_2_O as the minor SEI phase. Carbonate compounds were also detected, as indicated by the C 1s and O 1s spectra generated from carbonate electrolytes, as graphically described in Figure 3d.

After the stability test (see an inset picture in Figure 3d), a black-gray layer was grown on the surface, possibly indicating a high concentration of inactive lithium. Li species still be the main phase. Different from the sample after formation (Figure 3d,e), F and O species are also majorly presented through 200 nm, indicating that this lithium species stays in Li–F, Li–O, or Li–O–F species. On the contrary, Cu signal was only a minor observed species in this sample. For species fitting (Figure S13b: C 1s, Li 1s, and O 1s XPS narrow scans and fitting after the stability test), instead of other species also observed after the formation cycle, an outstanding thick layer of LiF was clearly detected, with a low degree of dead Li in the metal form. This indicates that a LiF-rich SEI can be beneficial for controlling Li plating, stripping, and reducing the level of dead Li in anode-free cells. However, a fresh Li surface is formed in every cycle, leading to the growth of this LiF layer, gradually consuming reversible Li^+^, as shown in a schematic diagram (Figure 3f). Therefore, there remains a trade-off in using fluorinated cosolvents in anode-free cells, particularly in practical configurations with a limited lithium source, requiring further research.

After a high-rate test, the white-gray layer formed after the formation cycle partially disappeared, and the Cu foil was clearly visible (see an inset photograph in Figure 3g). This sample most closely resembles bare Cu foil among the three samples. XPS and EDX mapping were also used to characterize surface chemistry. As shown in Figure 3g,h, lithium is also the main element on the Cu foil. However, according to the species fitting (Figure S13: C 1s, Li 1s, and O 1s XPS narrow scans and fitting after high rate), the main species was Li_2_O, and a clear LiF peak was observed after the formation cycle, getting thinner and only at the surface. This suggests that high-rate operation (up to 4C rate) can destroy the SEI of anode-free cells, as demonstrated graphically in Figure 3i. Moreover, as demonstrated in Figure S14 (Cu 2p XPS narrow scan), the signal of Cu^2+^ was clearly observed for the Cu foil after cycling but could not be detected after the formation and high-rate test, indicating that, after exposure to repeated lithium plating and stripping, corrosion of the Cu current collector is also another issue of anode-free cells.

To investigate structural evolution, *operando* XRD was conducted on a single-layer anode-free pouch cell and compared with a conventional NMC90/graphite full cell. Cylindrical cell analysis was not feasible due to thickness and the stainless-steel casing, so a pouch configuration with a Mo-source transmission mode was used to simultaneously characterize both electrodes (Figures 4a and S15). All pouch cells were cycled twice to ensure consistency for the Rietveld refinement. As shown in Figure S15, NMC90 and Cu and Al foils were detected in all samples, while only the graphite cell showed phase transitions (2H → LiC_12_ → LiC_6_), confirming the absence of graphite in anode-free cells.

**Figure 4:**
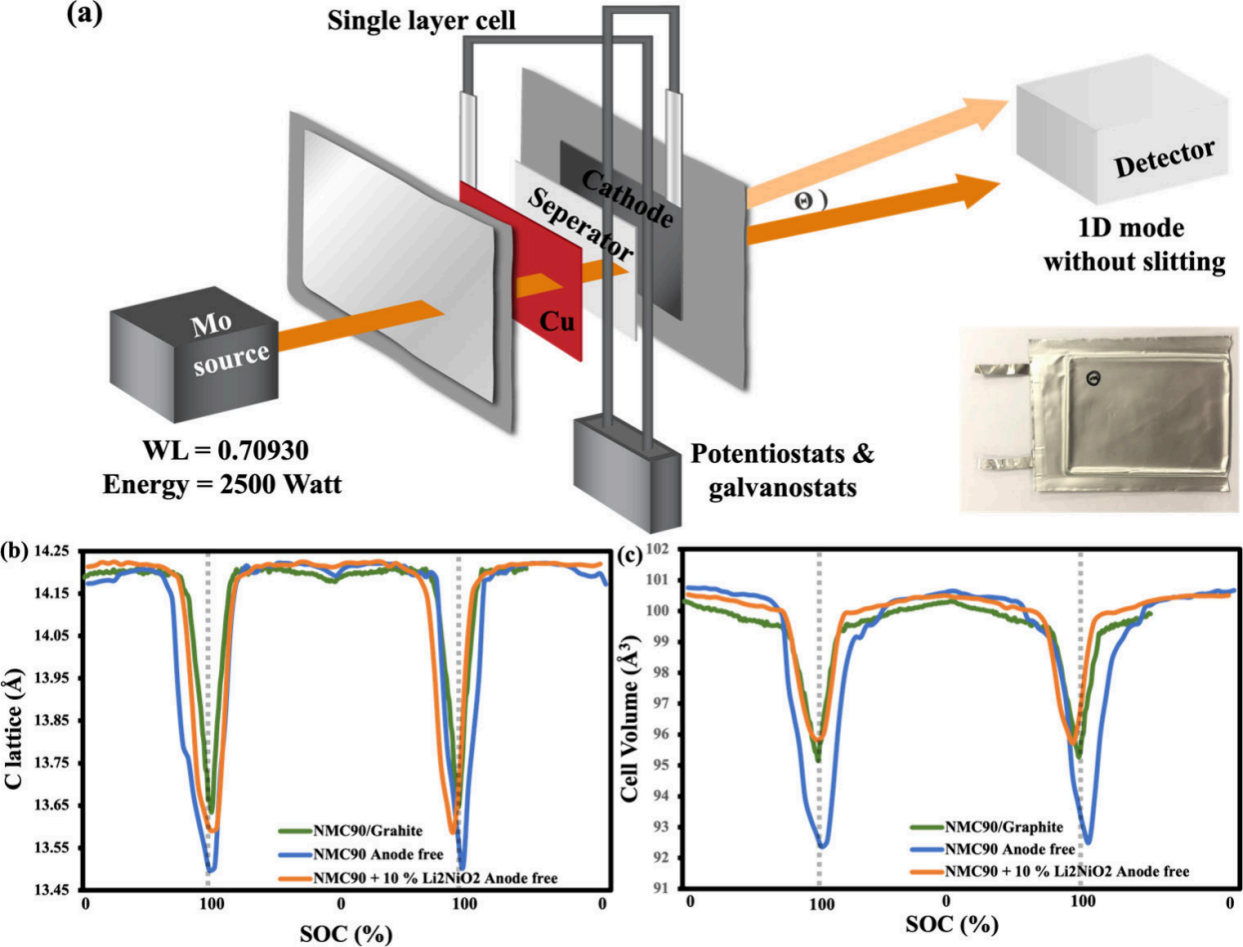
*Operando* XRD investigation of anode-free pouch cells: (a) schematic diagram of the experimental setup and small image of a pouch cell; (b) evolution of C-lattice parameters; (c) changes in unit cell volume during electrochemical cycling between 2.5 and 4.4 V.

For the cathode (Figure 4b,c), based on the (003), (101), and (104) planes (Figure S16), the anode-free cell showed greater changes in the lattice parameters and unit cell volume due to higher Li extraction, indicating Li loss during plating. These results highlight the importance of prelithiation. We used 10% Li_2_NiO_2_ in NMC90 due to its simple mixing process. As shown in Figure S17, NMC90 + 10 wt % Li_2_NiO_2_ exhibited reduced lattice changes, closer to those of the graphite cell, suggesting effective compensation of Li loss.

On the anode side (Figure S18), even at full charge (4.4 V), Li metal was not detected by *operando* XRD. A control test using commercial Li-plated Cu foil confirmed that crystalline Li metal gives a clear (110) peak at 2θ = 16.4°, which was absent in the pouch cell data. This suggests that Li plating possibly occurs in a noncrystalline or amorphous form. These findings underscore the challenge of characterizing plated lithium in anode-free cells, a critical factor for practical application, as discussed in the literature.^28^

In summary, this study demonstrates a significant advancement in the development of anode-free 18650 lithium-metal batteries by integrating 10 wt % Li_2_NiO_2_ as a prelithiation agent and employing a fluorine-rich electrolyte with 30% (v/v) FEC to stabilize lithium deposition. Our findings highlight that Li_2_NiO_2_ effectively compensates for initial lithium loss, thereby improving the Coulombic efficiency, while the use of a rigid stainless-steel casing with a tailored jelly roll configuration provides mechanical constraints that enhance cycling stability without requiring external pressure.

Post-mortem analysis reveals that lithium migration from the cathode to the copper current collector intensifies over extended cycling, contributing to performance degradation. Although the formation of a LiF-rich SEI mitigates inactive lithium accumulation, its effectiveness diminishes over prolonged operation due to continued lithium and electrolyte consumption. Furthermore, copper corrosion emerges as a newly identified degradation pathway, necessitating further research into electrolyte and current collector modifications to improve long-term stability.

Despite these challenges, our study successfully develops a commercial-scale 18650 AFLMB with an energy density of 320 Wh/kg, demonstrating stable cycling for 140 cycles and sustaining operation at a 4C rate. These findings provide a viable pathway for the commercialization of AFLMBs, particularly in high-energy-demand applications, such as military drones and electric mobility, where extended operational range and high energy density are critical. Future work will focus on refining electrolyte formulations and electrode architectures to further enhance the cycle life and minimize lithium depletion, paving the way for practical large-scale deployment of anode-free lithium-metal technology.

## Supplementary Material



## Notes

Experimental procedures for material synthesis, cell fabrication, and characterization methods, a minireview of related anode-free lithium-metal battery technologies, comprehensive battery performance checklist, detailed supporting results including material and cell specifications, electrochemical behavior, post-mortem analysis, and *operando* XRD investigation, and a supporting video (VDO S1) demonstrating the cylindrical cell winding process (PDF)
