# Supplementary material for: Stable Solid Electrolyte Interphase in Cylindrical Anode-Free Li-Metal NMC90 Batteries with Li2NiO2 Prelithiation and Fluorine-Rich Electrolytes for High Energy Density
Source: Nano Lett. 2025 May 22;25(22):9047–53. doi: 10.1021/acs.nanolett.5c01595 (PMC12142659; doi:10.1021/acs.nanolett.5c01595)
Supplement: Supplementary file 1 [file nl5c01595_si_001.pdf]

Supporting Information

Stable Solid Electrolyte Interphase in Cylindrical Anode-Free Li-Metal NMC90 Batteries with Li_2NiO_2 Pre-Lithiation and Fluorine-Rich Electrolytes for High Energy Density

Thitiphum Sangsanit, Ronnachai Songthan, Surat Prempluem, Worapol Tejangkura, and Montree Sawangphruk*

Centre of Excellence for Energy Storage Technology, Department of Chemical and Biomolecular Engineering, School of Energy Science and Engineering, Vidyasirimedhi Institute of Science and Technology, Rayong 21210, Thailand

*E-mail: montree.s@vistec.ac.th

Table of Contents

1. Experimental Procedures	S2
2. Mini review of related technologies	S3
3. Battery checklist	S5
4. Supporting results	S7
4.1 Production process	S7
4.2 Material and cell specifications	S7
4.3 Electrochemical behaviour	S11
4.4 Post-mortem analysis	S15
4.5 *Operando* XRD investigation of anode – free pouch cells	S18
5. Supporting VDO	S21

1.Experimental Procedures

Characterization of powdery materials and electrode

The NMC90 and Li-rich LNO (Li_2NiO_2) powder underwent examination using a field emission scanning electron microscope (FE-SEM, JEOL-JSM-7610F, JOEL Ltd.) equipped with a backscattered electron detector operating at an accelerating voltage of 15kV. Before the analysis, the powder samples were placed on carbon tape for direct characterization. To quantify the atomic composition, Wavelength Dispersive X-ray Fluorescence (WDXRF) was utilized. The crystallography of the powder was determined through powder XRD using a Bruker New D8 Advance diffractometer. Diffraction patterns in the range of 10° – 70° were obtained utilizing Cu-K alpha radiation.

Production of cylindrical cells

We procured a commercial NMC90 ($LiNi_{0.90}Co_{0.52}Mn_{0.48}O_2$) cathode, as detailed in Fig. S2 and Table S1, along with Li-rich LNO (Li_2NiO_2) demonstrated in Fig. S3, along with a binder and conductive carbon obtained from GELON (China). The cathode composition comprised 95.2 wt.% active mass (100% wt. NMC90 for NMC90 anode-free cell and 90% wt. NMC90 + 10% wt. Li_2NiO_2 for NMC90 + 10% Li_2NiO_2 anode-free cells), 2.4 wt.% polyvinylidene fluoride, and 2.4 wt.% carbon black (Super P). Copper foil with a thickness of 6.8 µm was sourced from MTI (China). The 18650 anode-free jelly rolls were rolled together through semi-automatic cylindrical cell winding, as illustrated in VDO S1. Battery-grade Li-salt ($LiPF_6$) and carbonate solvents (EC, DEC, EMC, and FEC) were obtained from GELON (China). The preparation of all electrolyte mixtures occurred in an MBRAUN (Germany) glove box under high-purity Ar gas. 18650 cells were assembled in a pilot plant under dry-room conditions, overseen by MTI (USA), with a dew point below -40 °C. Each cell contained approximately 4.5 g of electrolyte under all conditions. Following this, the completed cells underwent grading via IR_{AC} to assess the consistency of the production process.

Measurement of cylindrical cells

The assessment of 18650 full cell cycling and rate performance took place at room temperature utilizing a battery tester supplied by Neware (China). Preceding the evaluations, the cells experienced formation cycles at C/40 within the voltage range of 3.0 - 4.3 V to establish a stable solid electrolyte interface (SEI). To maintain uniformity and prevent biased selection, three samples from each experimental set were subjected to separate tests for cycling and rate performance. For cyclic performance assessment, the cells underwent testing with a charging rate of 0.5C and a discharge rate of 1.0C within the voltage range of 3.0 - 4.3 V. Regarding rate performance, the cells were charged at a rate of 0.5C and discharged at various rates, ranging from 0.1 to 4C.

Post-mortem analysis

Post-mortem electrodes were prepared by discharging and disassembling 18650 cells within an argon-filled glove box using an 18650 disassembly machine. Subsequently, they were cleaned with battery-grade dimethyl carbonate from GELON (China) to remove any residual Li-salt or solvent. The samples were then dried at a temperature of 25°C inside an MBRAUN (Germany) glove box for a duration of 12 h before characterization.

X-ray photoelectron spectroscopy (XPS, JPS-9010MC, JEOL) was employed for the analysis of Cu foil using an Mg-Kα radiation source (hυ = 1253.6 eV) at 12 kV and 25 mA under a high vacuum pressure of 10^{-7} Pa at room temperature. The aim was to compare the surface chemistry of Cu foil before and after testing. The XPS depth profile was conducted through Ar etching with 0 - 25 steps, each lasting 20 s. For the species fitting, the binding energy was verified by referencing adventitious carbon at 284.8 eV or the O 1s lithium oxide signal at 528.5 eV[1]. To prevent contamination or reactions with moisture and oxygen in the atmosphere, the disassembled samples were carefully transferred from the glove box to the XPS chamber using an air-sensitive holder. Furthermore, to analyze the cathode, XRD characterization was performed using a Bruker New D8 Advance diffractometer. Diffraction patterns in the range of 10°–90° were obtained utilizing Cu-K alpha radiation. Subsequently, Rietveld refinement with TOPAS software was applied to confirm both the cathode phase and lattice parameter.

Operando X-ray Diffraction

An *operando* XRD/electrochemistry approach was employed to investigate the behavior of a single-layer pouch cell during charging and discharging. The setup involved connecting the cell to a D8 Advance XRD machine and a Metrohm Autolab electrochemical workstation (PGSTAT 302 N), as depicted in Fig S15. Diffraction patterns were collected at intervals of 1 second per step within the 2-theta range of 5°–50° using Mo Ka-radiation with a wavelength (WL) of 0.70930 and 2500 Watt energy. Charging and discharging operations were carried out between 2.5 V and 4.4 V for two cycles at C/20. Subsequently, the gathered data were analyzed using the Rietveld refinement method with TOPAS software version 5.0 (Bruker AXS).

References

(1) Otto, S.-K.; Moryson, Y.; Krauskopf, T.; Peppler, K.; Sann, J.; Janek, J.; Henss, A. In-Depth Characterization of Lithium-Metal Surfaces with XPS and ToF-SIMS: Toward Better Understanding of the Passivation Layer. *Chemistry of Materials* **2021**, *33* (3), 859-867. DOI: 10.1021/acs.chemmater.0c03518. Wood, K. N.; Teeter, G. XPS on Li-Battery-Related Compounds: Analysis of Inorganic SEI Phases and a Methodology for Charge Correction. *ACS Applied Energy Materials* **2018**, *1* (9), 4493-4504. DOI: 10.1021/acsaem.8b00406. Menkin, S.; O'Keefe, C. A.; Gunnarsdóttir, A. B.; Dey, S.; Pesci, F. M.; Shen, Z.; Aguadero, A.; Grey, C. P. Toward an Understanding of SEI Formation and Lithium Plating on Copper in Anode-Free Batteries. *The Journal of Physical Chemistry C* **2021**, *125* (30), 16719-16732. DOI: 10.1021/acs.jpcc.1c03877.

2. Mini review of related technologies

Table S1. The parameters of recently published literatures focused on developing anode-free lithium batteries

Cathode	Electrolyte	Scale	External pressure	Performance	Ref.
NMC622	1M $LiPF_6$ + EC/DEC/EMC + 5% FEC v/v	PEEK cell (modified coin cell)	1 MPa	70.5% retention after 200 cycles at 0.2C charge 0.5C discharge	[1]
$LiNi_{0.5}Mn_{1.5}O$	4.5 M LiFSI salt in Py13FSI with 1 wt% LiTFSI	Coin cell	-	> 80% retention after 120 cycles at 1C	[2]
$LiMn_2O_4$	1 M $LiPF_6$ + 0.2 0.02 M LiDFOB in FEC/HFE/FEMC in 2:2:6 mass ratio	Coin cell	-	83.3 % retention after 40 cycles at 1C	[3]
NMC532	2.0 M LiDFOB + 1.4 M $LiBF_4$ in FEC:DEC 1:2 v/v	Pouch cell	1,170 kPa	> 89 % retention after 200 cycles at 0.2C charge 0.5C discharge	[4]
NMC532, NMC811, LCO, LFP	0.6 M LiDFOB + 0.6 M $LiBF_4$ in FEC:DEC 1:2 v/v	Pouch cell	200 kPa – 1,200 kPa	Variations occur among different cathodes and pressure levels.	[5]
NMC532	0.6 M LiDFOB + 0.6 M $LiBF_4$ in FEC:DEC 1:2 v/v	Pouch cell	1,200 kPa	> 90 % retention after 50 cycles at 1C	[6]
NMC811	LiFSI in DME, and a highly fluorinated ether (HFE) in a molar ratio of 1:1.2:2	Pouch cell	20 psi	> 85 % retention after 200 cycles at 0.1C	[7]
NMC532	1 M $LiPF_6$ FEC:DEC 1:2 v/v and 1M $LiPF_6$ FEC:TFEC 1:2 v/v	Pouch cell	1,200 kPa	> 50 % retention after 80 cycles at 0.1C	[8]
Li–Cu	4.6 M LiFSI + 2.3 M LiTSI in DME	Pouch cell	350 kPa	-	[9]

Cathode	Electrolyte	Scale	External pressure	Performance	Ref.
NMC811	1M $LiPF_6$ in EC/DEC 1:1 v/v	Coin cell, Pouch cell	1200 kPa	87.3 % retention after 20 cycles at 0.2C charge 1C discharge in coin cell	[10]
Li–Cu	Simulation	Simulation	744 kPa – 1585 kPa		[11]
NMC90 + 10 % Li_2NiO_2	1.2 M $LiPF_6$ in ECDECEMCFEC 1:1:1:1.3 v/v	Cylindrical cell	-	> 40 % retention after 100 cycles at 0.5 C charge 1 C discharge	This work

References for Table S1

1. Liu W, Luo Y, Hu Y, Chen Z, Wang Q, Chen Y, et al. Interrelation Between External Pressure, SEI Structure, and Electrodeposit Morphology in an Anode-Free Lithium Metal Battery. **Advanced Energy Materials**. 2024;14(5):2302261.

2. Liang P, Sun H, Huang C-L, Zhu G, Tai H-C, Li J, et al. A Nonflammable High-Voltage 4.7 V Anode-Free Lithium Battery. **Advanced Materials**. 2022;34(51):2207361.

3. Chen L, Chiang C-L, Zeng G, Tang Y, Wu X, Zhou S, et al. Enhancing the cycle-life of initial-anode-free lithium-metal batteries by pre-lithiation in Mn-based Li-rich spinel cathodes. **Journal of Materials Chemistry A**. 2023;11(21):11119-25.

4. Louli AJ, Eldesoky A, Weber R, Genovese M, Coon M, deGooyer J, et al. Diagnosing and correcting anode-free cell failure via electrolyte and morphological analysis. **Nature Energy**. 2020;5(9):693-702.

5. Louli AJ, Eldesoky A, deGooyer J, Coon M, Aiken CP, Simunovic Z, et al. Different Positive Electrodes for Anode-Free Lithium Metal Cells. **Journal of The Electrochemical Society**. 2022;169(4):040517.

6. Weber R, Genovese M, Louli AJ, Hames S, Martin C, Hill IG, et al. Long cycle life and dendrite-free lithium morphology in anode-free lithium pouch cells enabled by a dual-salt liquid electrolyte. **Nature Energy**. 2019;4(8):683-9.

7. Lim H-S, Nguyen DT, Lochala JA, Cao X, Zhang J-G. Improving Cycling Performance of Anode-Free Lithium Batteries by Pressure and Voltage Control. **ACS Energy Letters**. 2024;9(1):126-35.

8. Louli AJ, Genovese M, Weber R, Hames SG, Logan ER, Dahn JR. Exploring the Impact of Mechanical Pressure on the Performance of Anode-Free Lithium Metal Cells. **Journal of The Electrochemical Society**. 2019;166(8):A1291.

9. Fang C, Lu B, Pawar G, Zhang M, Cheng D, Chen S, et al. Pressure-tailored lithium deposition and dissolution in lithium metal batteries. **Nature Energy**. 2021;6(10):987-94.

10. Zhou C, Samson AJ, Garakani MA, Thangadurai V. Communication—Anode-Free Lithium Metal Batteries: A Case Study of Compression Effects on Coin Cell Performance. **Journal of The Electrochemical Society**. 2021;168(6):060532.

11. Zhang X, Wang QJ, Harrison KL, Jungjohann K, Boyce BL, Roberts SA, et al. Rethinking How External Pressure Can Suppress Dendrites in Lithium Metal Batteries. **Journal of The Electrochemical Society**. 2019;166(15):A3639.

3. Battery checklist

Table S2. Checklist for battery-related literature.
This checklist was adapted from the battery research field's battery checklist[1, 2].

Table I. Information Checklist

**Electrodes and Cells**	
**Cell type and configuration**	18650 anode free configuration
**Electrodes' geometry and size**	Cathode: length 106 cm. width 5.8 cm.<br>Anode: length 111 cm. width 5.8 cm.
**Description of electrode preparation (including post-coating treatments such as compression/calendering)**	Cathode mixing ratio: 95.2: 2.4: 2.4 active mass: Super P: PVDF<br>at 70 - 2,000 rpm for 12 hours.<br>Solvent evaporation: 145 °C<br>Calendering force: 6 tons
**Electrode's active material areal mass loading (or areal capacity)**	Cathode: 18.03 ± 1.43 g/electrode<br>~ 29.3 mg/cm² for double-sided coating and 14.7 mg/cm² for single-sided coating.
**Composition of the electrodes, including supplier and purity of pursued components**	95.2 wt.% Active materials: NMC90 and Li_2NiO_2 from Gelon (China)<br>2.4 wt.% Binder: PVDF from Gelon (China)<br>2.4 wt.% Conductive additive: Super P from Gelon (China)<br>Battery grade<br>(< 20 PPM moisture impurity)
**Apparent electrode density (calculated as the ratio of areal mass loading and thickness)**	1.93 g/cm^3
**Current collector type and thickness (if not flat, also weight)**	Cathode: Al foil with 14 um thickness<br>Anode: Cu foil with 6.8 um thickness
**Separator type & thickness**	Al_2O_3 coated PP/PE/PP & 16 um from Yunji (China)
**Electrolyte composition and volume/weight used in the cell**	1.2 M $LiPF_6$ EC:DEC:EMC:FEC (1:1:1:1.3, v/v)<br>4.68 ± 0.14 g per 18650 cell
**Electrochemical testing**	
**Testing temperature**	25°C
**Voltage (or potential) range**	Formation cycle: 3.0 – 4.4 V<br>Stability and rate performance: 3.0 – 4.3 V
**Theoretical capacity and specified C-rate**	1 C = 3,400 mAh/cell,

	200 mAh/g of NMC
**Charge / discharge program (CC, CV, combination)**	CCCV charge and CC discharge
**C-rate for each electrochemical measurement**	From 0.1 – 4 C rate
**Initial electrochemical profile**	82.88 ± 0.72% initial coulombic efficiency (ICE)
**Cycling performance**	20 % after 140 cycles
**Coulombic efficiency associated with cycling data**	Approaching 100 %

Table II. Performance Reporting

**Cell type**		Coin cell		Pouch cell		Other ☑
**Cell configuration**		2-electrode cell ☑		3-electrode cell		
		Coin cell		Pouch cell		Other
**Mass loading of active material**	<2 mg cm^{-2}					
	2-5 mg cm^{-2}					
	5-8 mg cm^{-2}					
	>8 mg cm^{-2}					Cathode: 18.03 ± 1.43 g/electrode ~ 29.3 mg/cm² for double-sided coating and 14.7 mg/cm² for single-sided coating.
**Number of cycles at ≤1C**		Metal anode/battery (incl. half cells)		Full cell		
	<50					
	50-200			Capacity Retention: 20% after 140 cycles, tested at 0.5C CCCV charge and 1C CC discharge rates.		
	>200					

References for Table S2

1. Li J, Arbizzani C, Kjelstrup S, Xiao J, Xia Y-y, Yu Y, et al. Good practice guide for papers on batteries for the Journal of Power Sources. **Journal of Power Sources**. 2020;452:227824.
2. Stephan AK. Standardized Battery Reporting Guidelines. **Joule**. 2021;5(1):1-2.

4. Supporting Results and Discussion

4.1 Production process

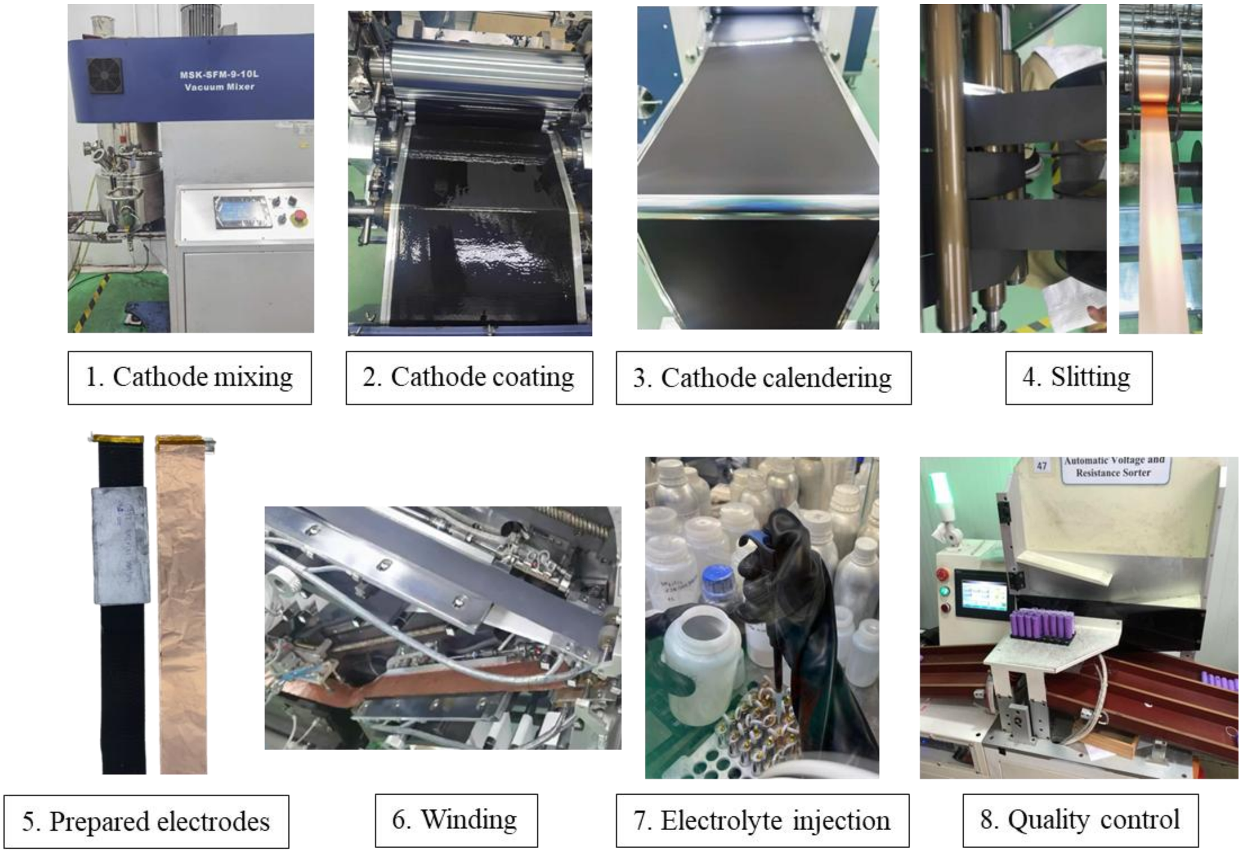

Fig. S1. Anode-free 18650 cylindrical cell production process at the pilot plant scale**.**

4.2 Materials and cell specifications

Table S3. Chemical composition of NMC90 determined by WDXRF.

Sample	Elemental (at. %)		
	Ni	Co	Mn
NMC powder	90.00	5.20	4.80

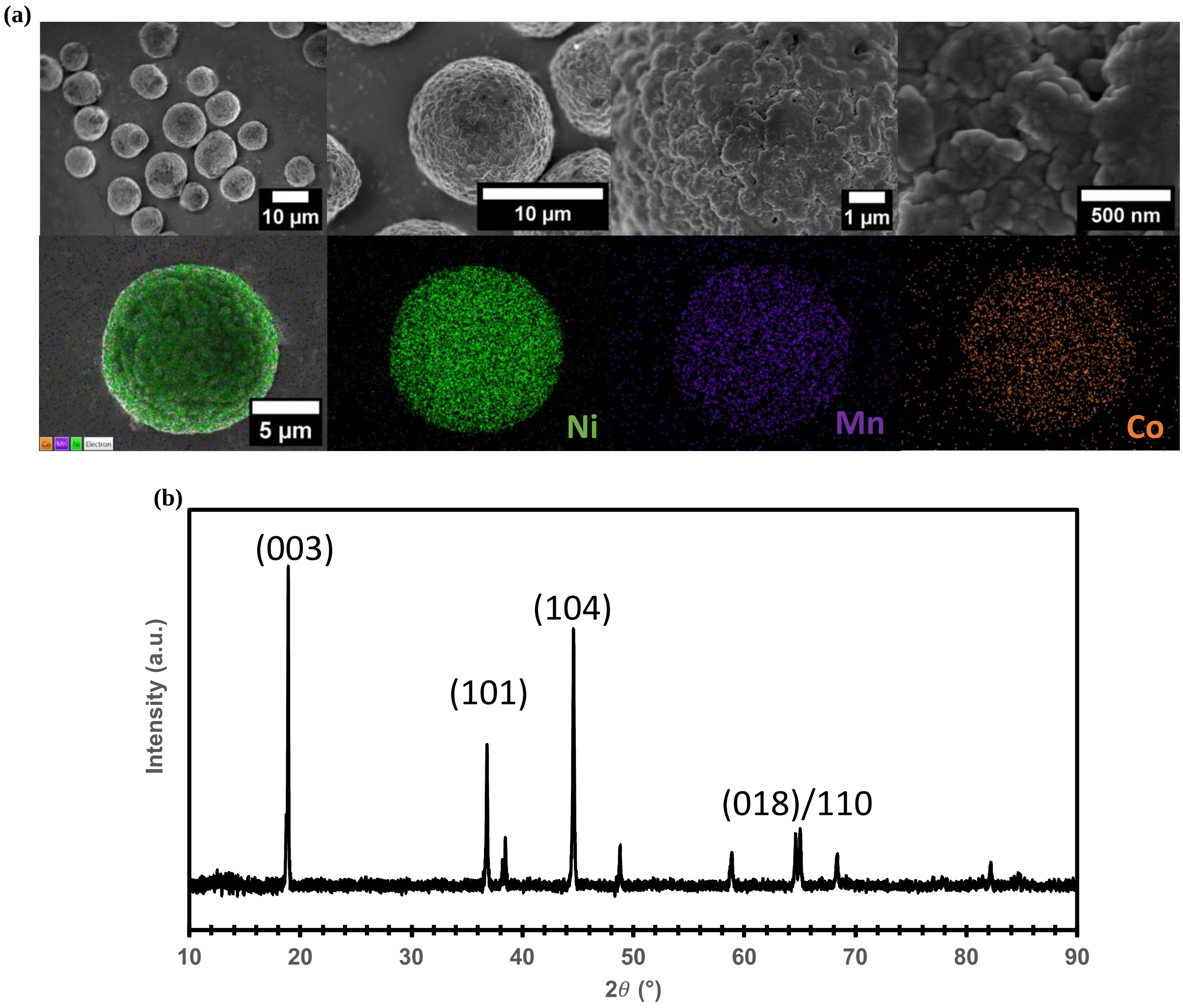

Sample	SG	a(Å)	c(Å)	C/A	I_{003}/I_{104}	R_{Bragg}
NMC90	R3-m	2.8703(0)	14.1797(7)	4.94	1.469	1.17

Fig. S2. Characterization of NMC90 powder: (a) FESEM images and (b) powder X-ray diffraction (XRD).

(a)

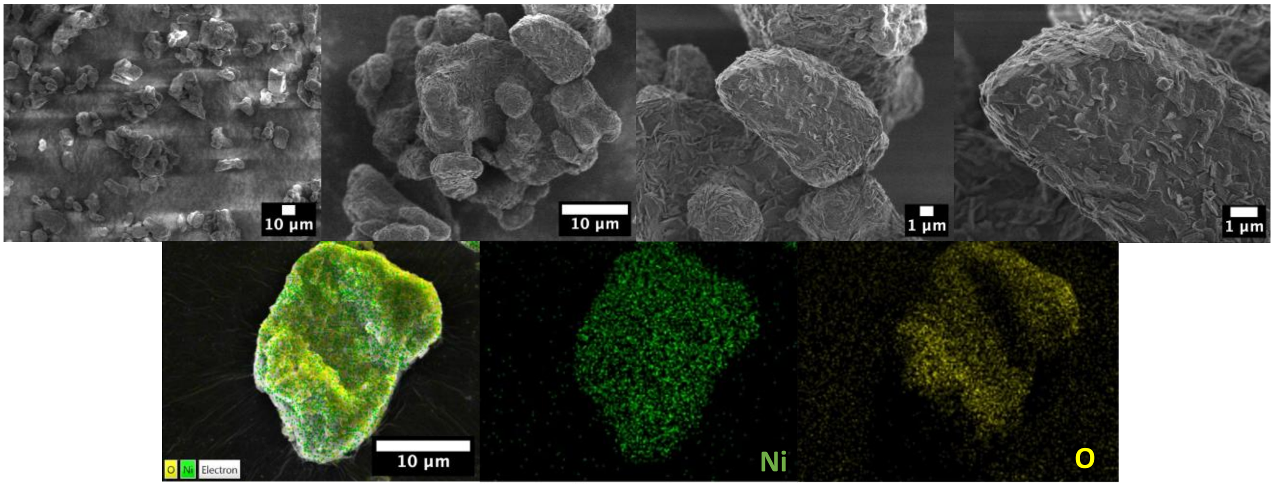

(b)

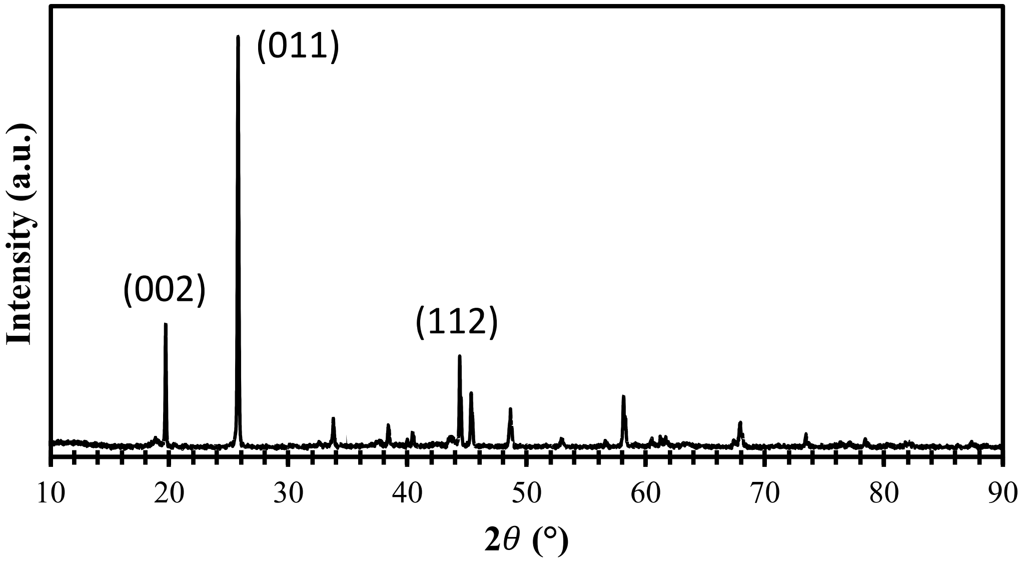

Sample	SG	a(Å)	b(Å)	c(Å)	R_{Bragg}
Li_2NiO_2	Immm	2.8886(5)	3.7474(6)	9.0317(2)	2.46

Fig. S3. Characterization of Li_2NiO_2 powder: (a) FESEM images and (b) powder X-ray diffraction (XRD).

Table S4. Properties of electrode and cells of anode free NMC90 + 10 % Li_2NiO_2 18650 cells

**Electrode properties**	**Anode: Cu foil**	Coating thickness = 0 μm<br>Electrode thickness = 6.8 μm<br>Length = 111 cm.
	**Cathode: NMC90 + 10 % Li_2NiO_2**	Coating thickness = 214 μm<br>Electrode thickness = 152 ± 7.21 μm<br>(Pressing 6 tons)<br>Length = 106 cm.<br>Active mass = 18.03 ± 1.43 g
**N/P ratio**	-	
**Weight**	40.51 ± 1.18 g (n = 7)	
**Electrolyte**	1.2 M $LiPF_6$ EC:DEC:EMC:FEC (1:1:1:1.3, v/v) 4.68 ± 0.14 g (n = 7)	
**Capacity**	3,407.30 ± 115.15 mAh (0.1C) 3,284.6 ± 103.90 mAh (0.2 C)	
**Nominal voltage**	3.81 V	
**Energy density**	Volumetric	811 Wh/L $_{(16\ ml).}$
	Gravimetric	320 Wh/kg_{cell}
		**510 Wh/$kg_{jelly\ (25.44\ g)}$**
		695 Wh/$kg_{active\ mass.}$
**Internal resistance**	Before formation: 72.71 ± 6.07 mΩ, (n = 7)<br>After formation: 54.14 ± 2.54 mΩ, (n = 7)	

4.3 Electrochemical behavior

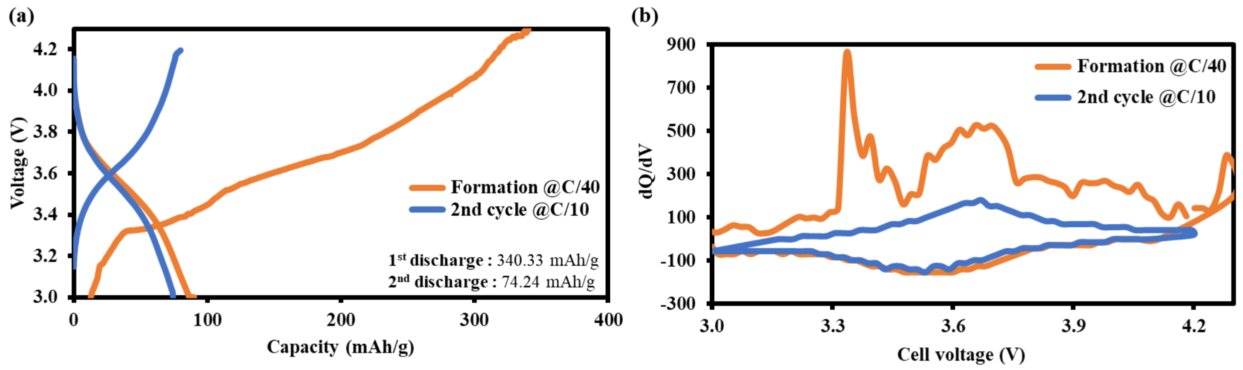

Fig. S4. Electrochemical behavior of Li_2NiO_2 18650 cells with 1.2 M $LiPF_6$ EC:DEC:EMC:FEC (1:1:1:1.3, v/v) (a) charge-discharge profile and (b) different capacity as a function of cell voltage.

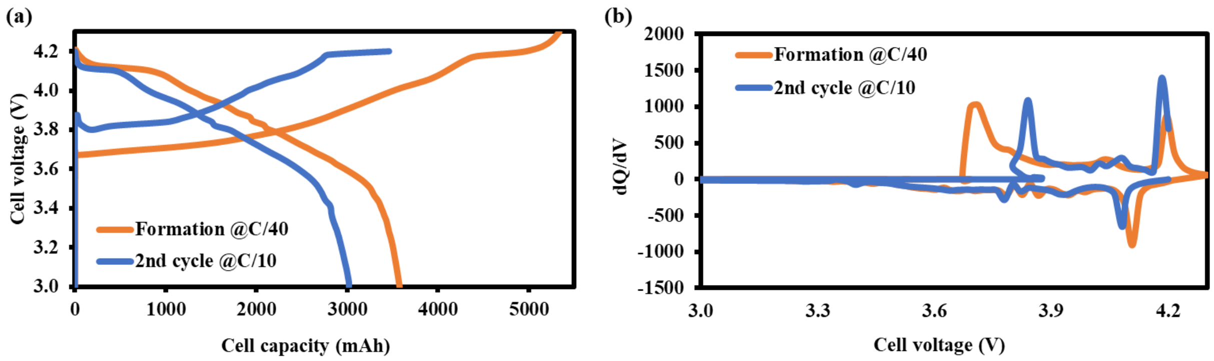

Fig. S5. Electrochemical behavior of anode free NMC90 18650 cells with 1.2 M $LiPF_6$ EC:DEC:EMC:FEC (1:1:1:1.3, v/v) (a) charge-discharge profile and (b) different capacity as a function of cell voltage.

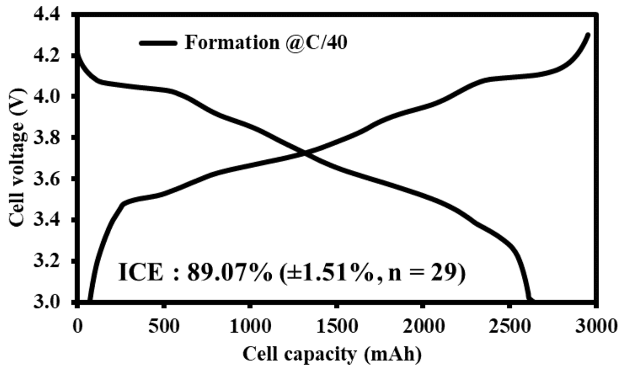

Fig. S6. Charge-discharge profile of formation cycle for graphite/NMC90 18650 cells with 1.2 M $LiPF_6$ EC:DEC:EMC:FEC (1:1:1:1.3, v/v).

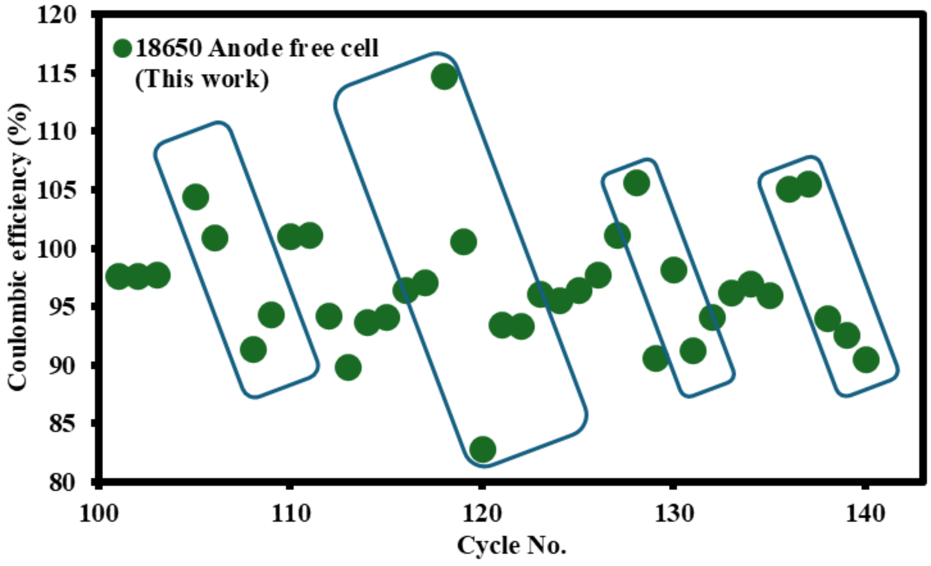

Fig. S7. Coulombic efficiency of the 18650 NMC90 + 10% Li_2NiO_2 anode-free cell over the first 100 cycles.

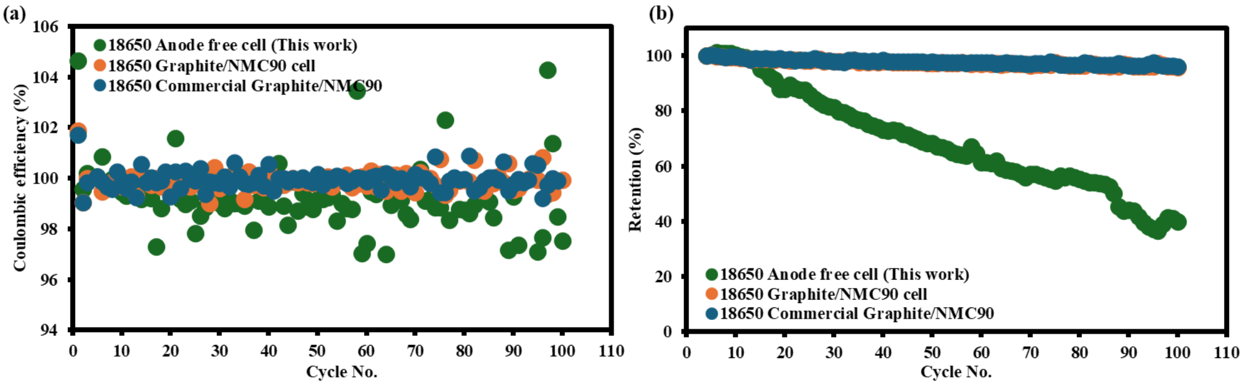

Fig. S8. Electrochemical behavior of 18650 NMC90 + 10% Li_2NiO_2 anode-free cell compared to in-house graphite//NMC90 and commercial graphite//NMC cells: (a) Coulombic efficiency and (b) capacity retention over the first 100 cycles.

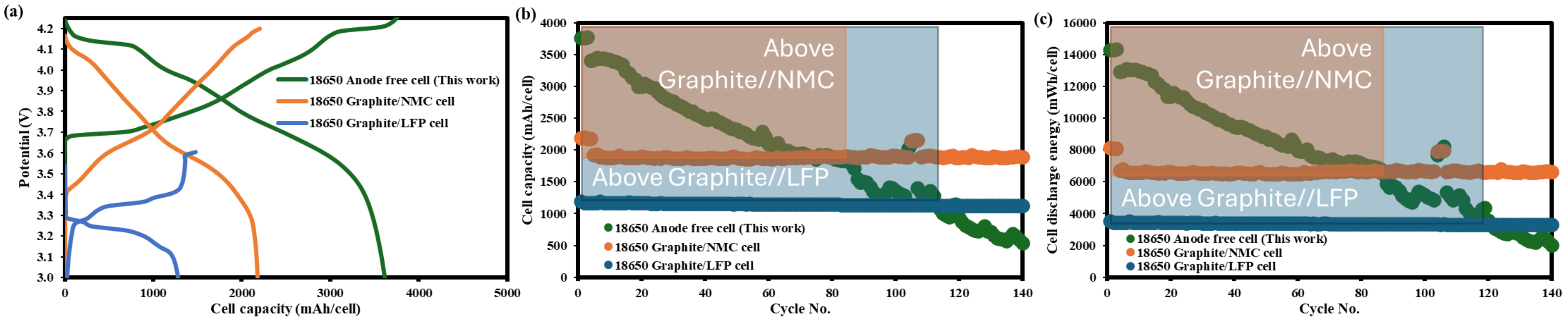

Fig. S9. Cell performance of the 18650 NMC90 + 10% Li_2NiO_2 anode-free cell compared to in-house graphite//NMC90 and commercial graphite//NMC cells: (a) initial charge–discharge profile at 0.1C, (b) cell capacity retention, and (c) cell discharge energy retention at 0.5C charge and 1C discharge.

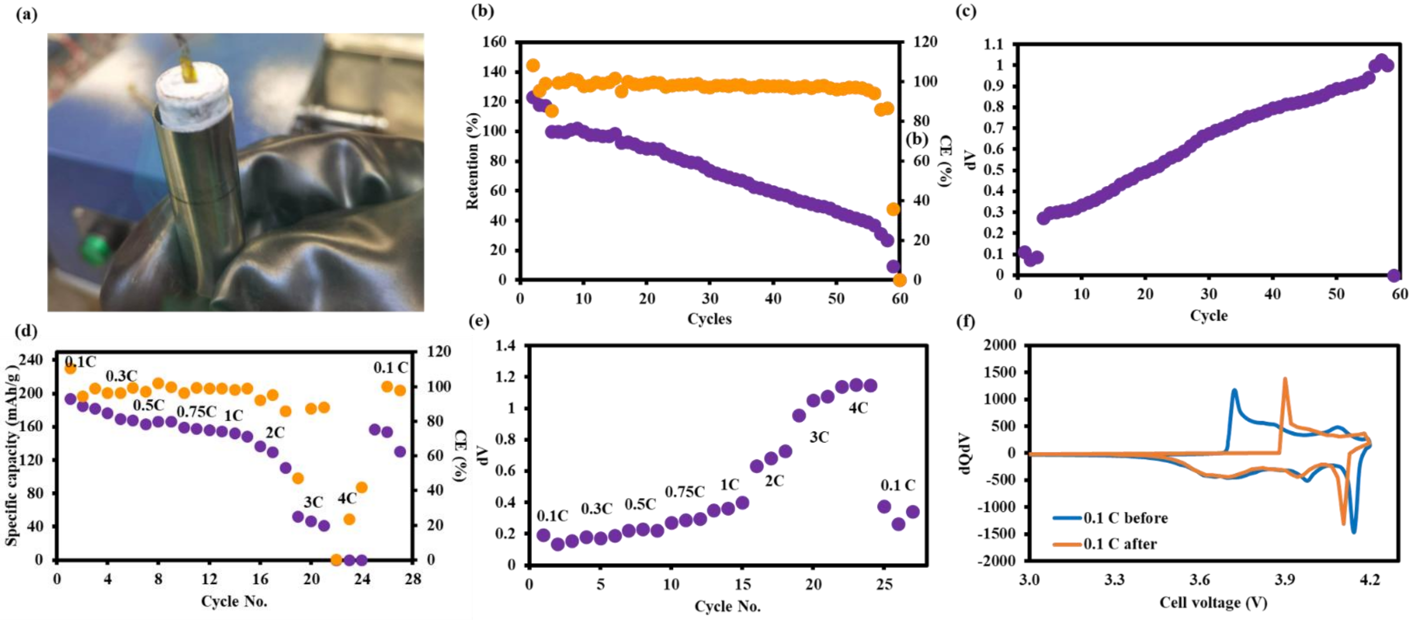

Fig. S10. (a) Image of unfitted jelly roll. (b-f) Electrochemical behavior for unfitted jelly roll anode-free NMC90 + 10% Li_2NiO_2 with 1.2 M $LiPF_6$ EC:DEC:EMC:FEC (1:1:1:1.3, v/v): (a-c) Cycling performance at 0.5C charge, 1C discharge between 3.0 – 4.3 V: (b) Capacity retention. (c) Voltage change (dV). (d-f) Rate performance: (d) Specific capacity. (e) Voltage change (dV). (f) Different capacity as a function of cell voltage before and after testing at different discharge C-rates (1 C = 2,200 mA).

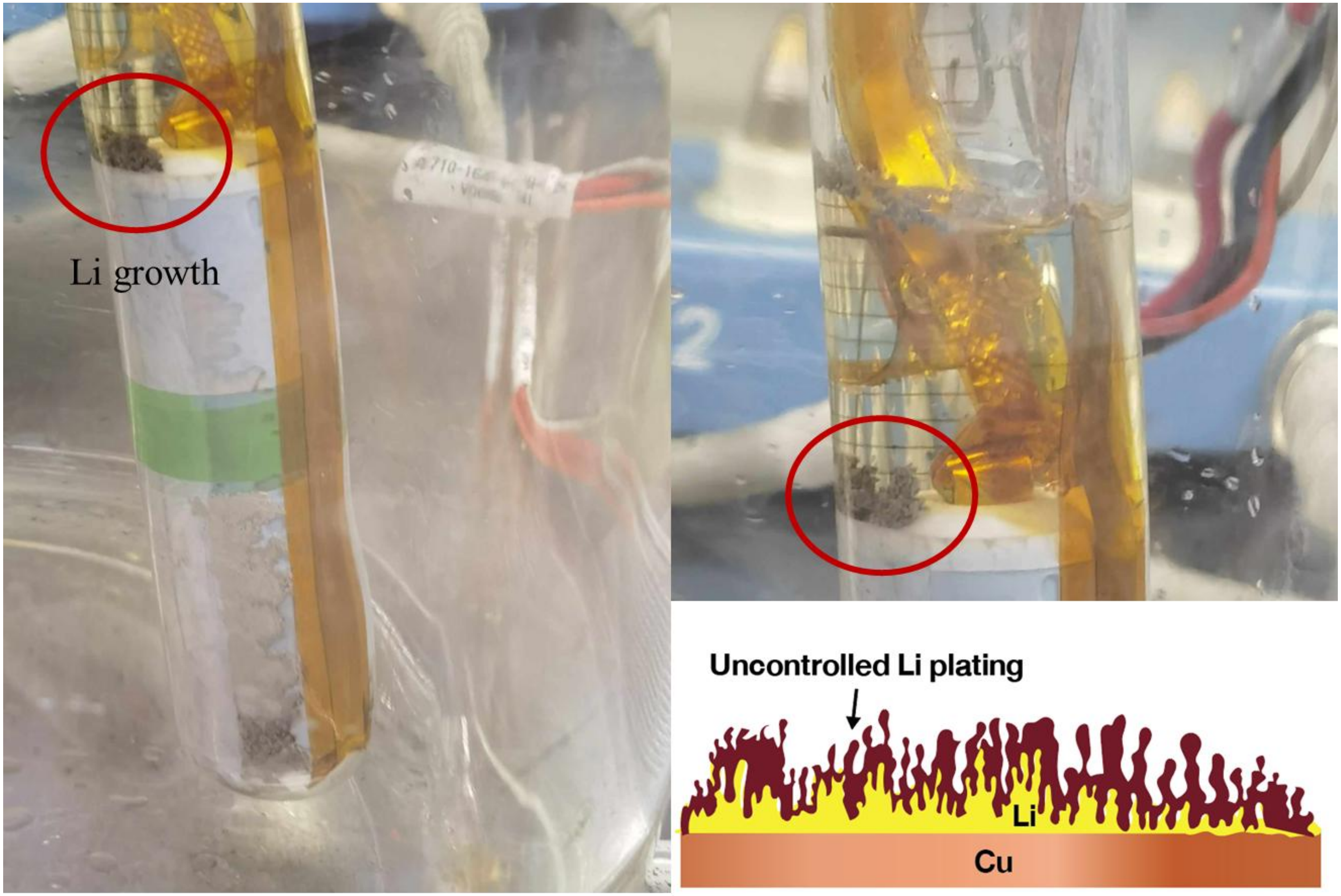

Fig. S11. Anode free NMC90 + 10 % L_2NiO_2 jelly roll in mimic 18650 glass cell during cycling inside an argon-filled glove box.

4.4 Post-mortem analysis

(a)

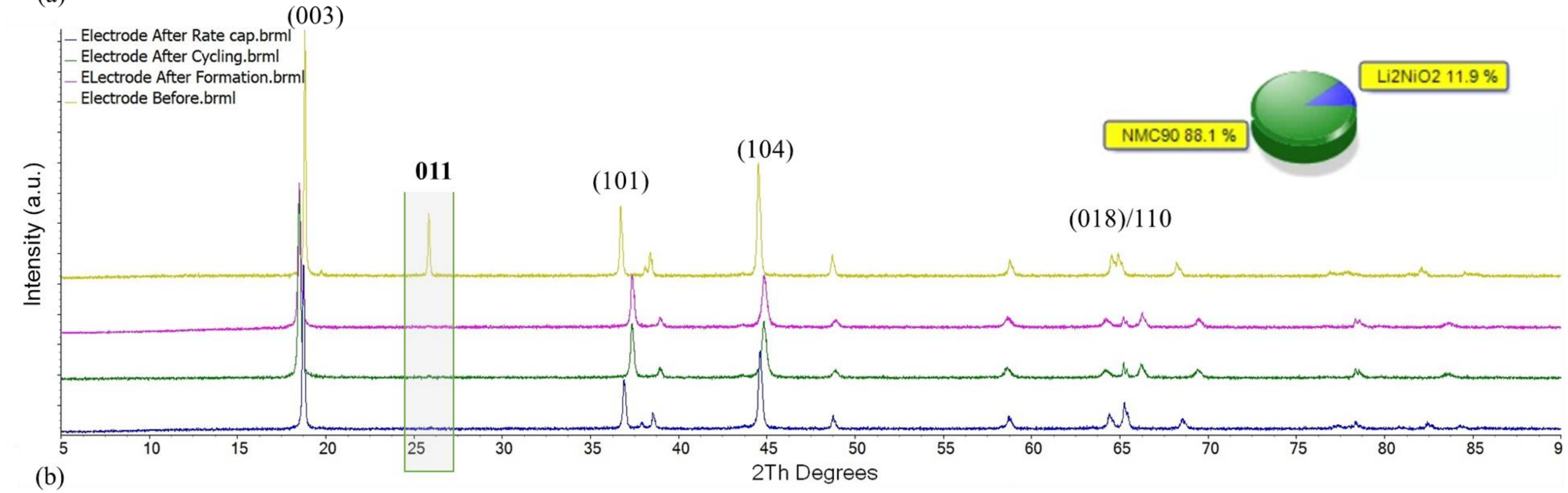

(b)

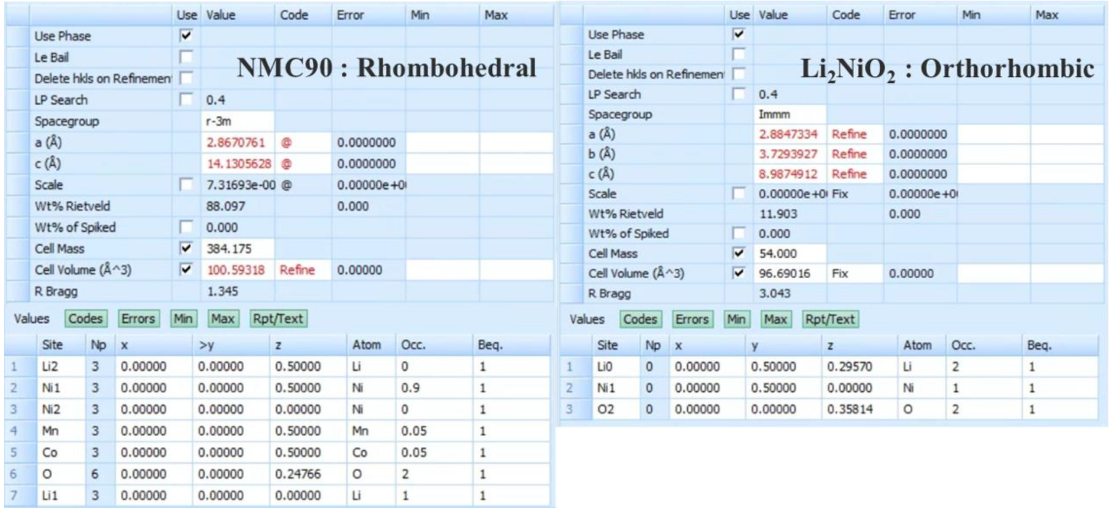

NMC90 : Rhombohedral

	Use	Value	Code	Error	Min	Max
Use Phase	✓					
Le Bail	☐					
Delete hkls on Refinemen	☐					
LP Search	☐	0.4				
Spacegroup		r-3m				
a (Å)		2.8670761	@	0.0000000		
c (Å)		14.1305628	@	0.0000000		
Scale	☐	7.31693e-00	@	0.00000e+0		
Wt% Rietveld		88.097		0.000		
Wt% of Spiked	☐	0.000				
Cell Mass	✓	384.175				
Cell Volume (Å^3)	✓	100.59318	Refine	0.00000		
R Bragg		1.345				

Values | Codes | Errors | Min | Max | Rpt/Text

	Site	Np	x	>y	z	Atom	Occ.	Beq.
1	Li2	3	0.00000	0.00000	0.50000	Li	0	1
2	Ni1	3	0.00000	0.00000	0.50000	Ni	0.9	1
3	Ni2	3	0.00000	0.00000	0.00000	Ni	0	1
4	Mn	3	0.00000	0.00000	0.50000	Mn	0.05	1
5	Co	3	0.00000	0.00000	0.50000	Co	0.05	1
6	O	6	0.00000	0.00000	0.24766	O	2	1
7	Li1	3	0.00000	0.00000	0.00000	Li	1	1

Li_2NiO_2 : Orthorhombic

	Use	Value	Code	Error	Min	Max
Use Phase	✓					
Le Bail	☐					
Delete hkls on Refinemen	☐					
LP Search	☐	0.4				
Spacegroup		Immm				
a (Å)		2.8847334	Refine	0.0000000		
b (Å)		3.7293927	Refine	0.0000000		
c (Å)		8.9874912	Refine	0.0000000		
Scale	☐	0.00000e+0	Fix	0.00000e+0		
Wt% Rietveld		11.903		0.000		
Wt% of Spiked	☐	0.000				
Cell Mass	✓	54.000				
Cell Volume (Å^3)	✓	96.69016	Fix	0.00000		
R Bragg		3.043				

Values | Codes | Errors | Min | Max | Rpt/Text

	Site	Np	x	y	z	Atom	Occ.	Beq.
1	Li0	0	0.00000	0.50000	0.29570	Li	2	1
2	Ni1	0	0.00000	0.50000	0.00000	Ni	1	1
3	O2	0	0.00000	0.00000	0.35814	O	2	1

(c)

**Samples**	**Phase Name**	**SG**	**a (Å)**	**c (Å)**
Fresh electrode	NMC90	r-3m	2.86708	14.13057
After Formation	NMC90	r-3m	2.817615	14.36245
After Cycling	NMC90	r-3m	2.818915	14.36859
After High rate	NMC90	r-3m	2.853603	14.18557

Fig. S12. XRD Characterization of NMC90 + 10% Li_2NiO_2 electrode: (a) XRD pattern (b) properties of fitting species for Rietveld refinement and (c) lattice parameter of the main phase in NMC90.

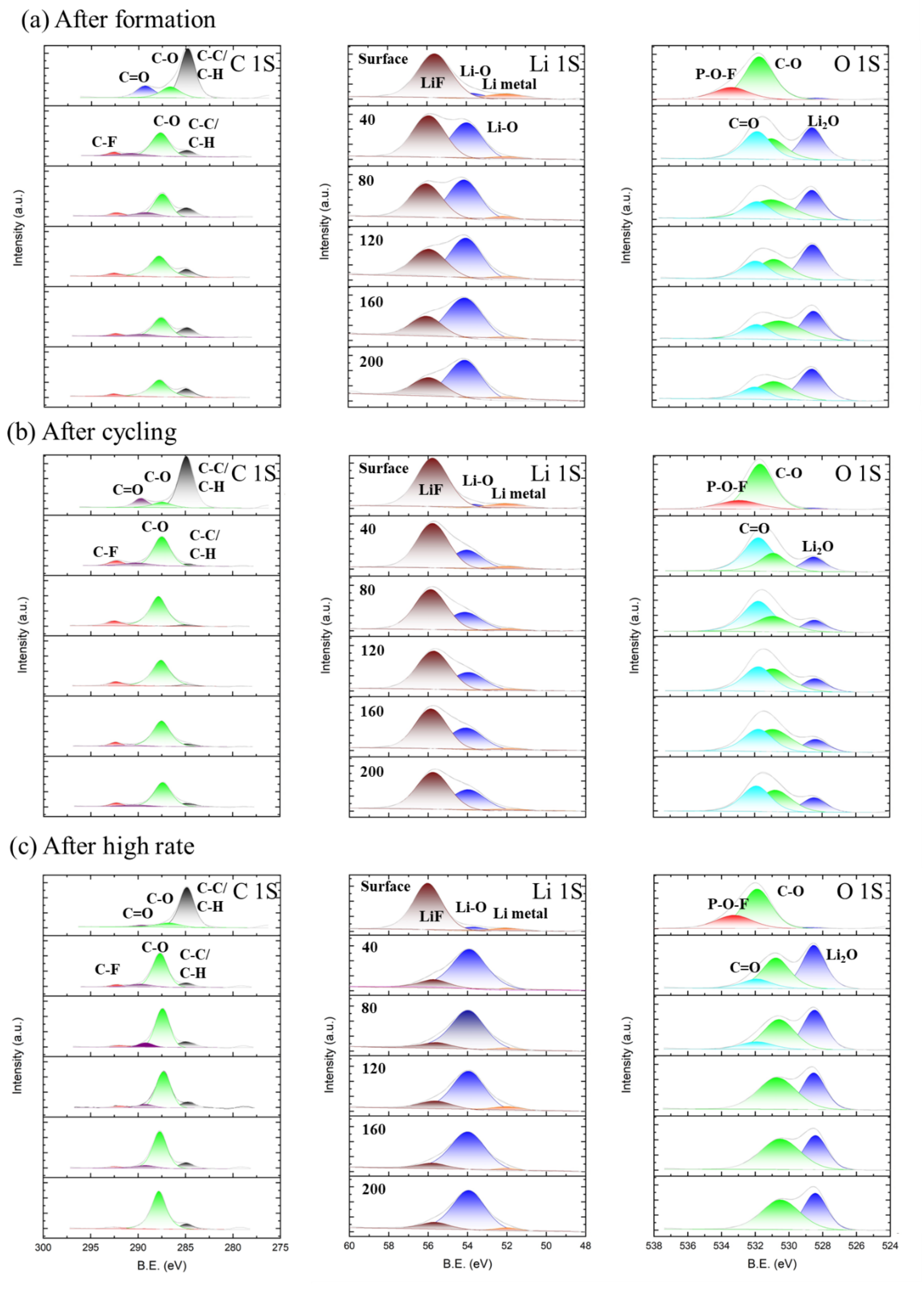

Fig. S13. XPS species fitting of NMC90 + 10% Li_2NiO_2 electrode: (a) after formation at C/40 (b) after 140 cycles at 0.5 C charge 1.0 C discharge and (c) after high C-rate up to 4 C-rate.

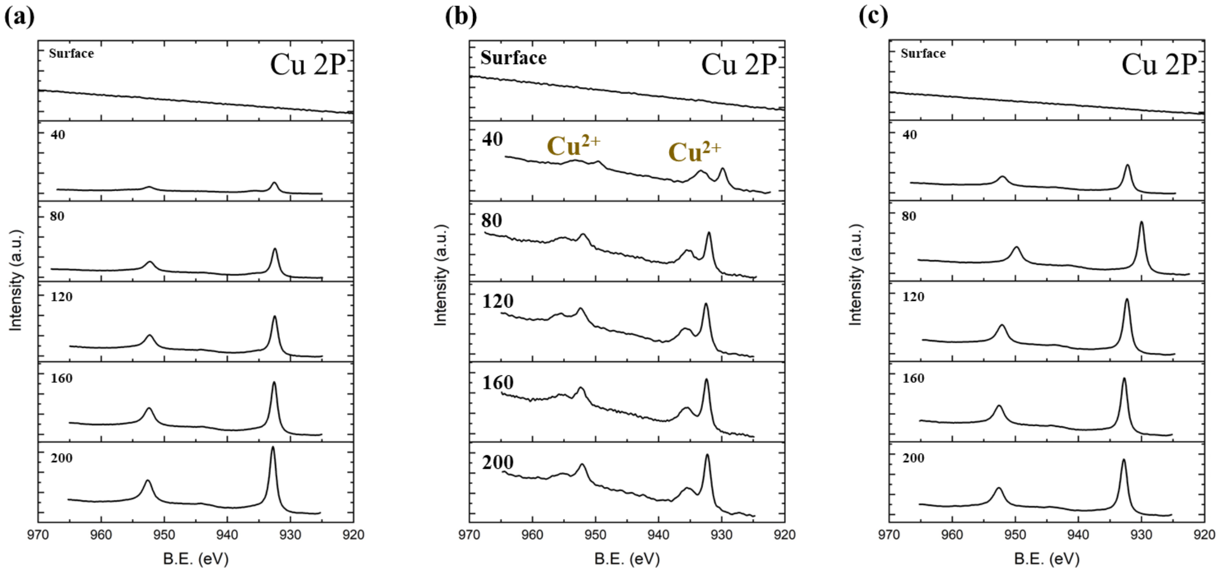

Fig. S14. Cu 2P XPS of NMC90 + 10% Li_2NiO_2 electrode: (a) after formation at C/40 (b) after 140 cycles at 0.5 C charge 1.0 C discharge and (c) after high C-rate up to 4 C-rate.

4.5 *Operando* XRD investigation of anode – free pouch cells

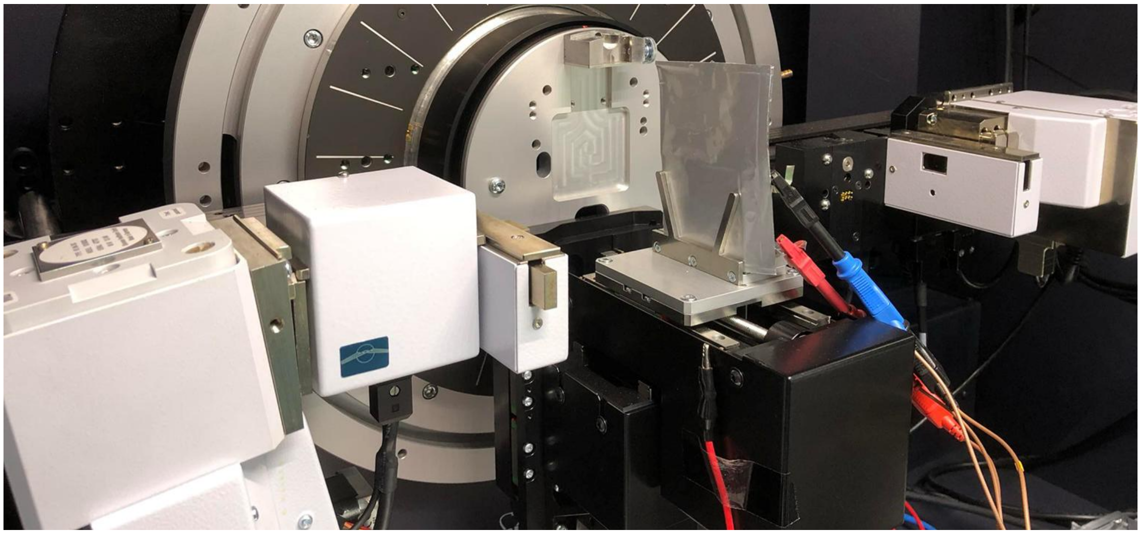

Fig. S15. Image of *operando* XRD set-up for pouch cell.

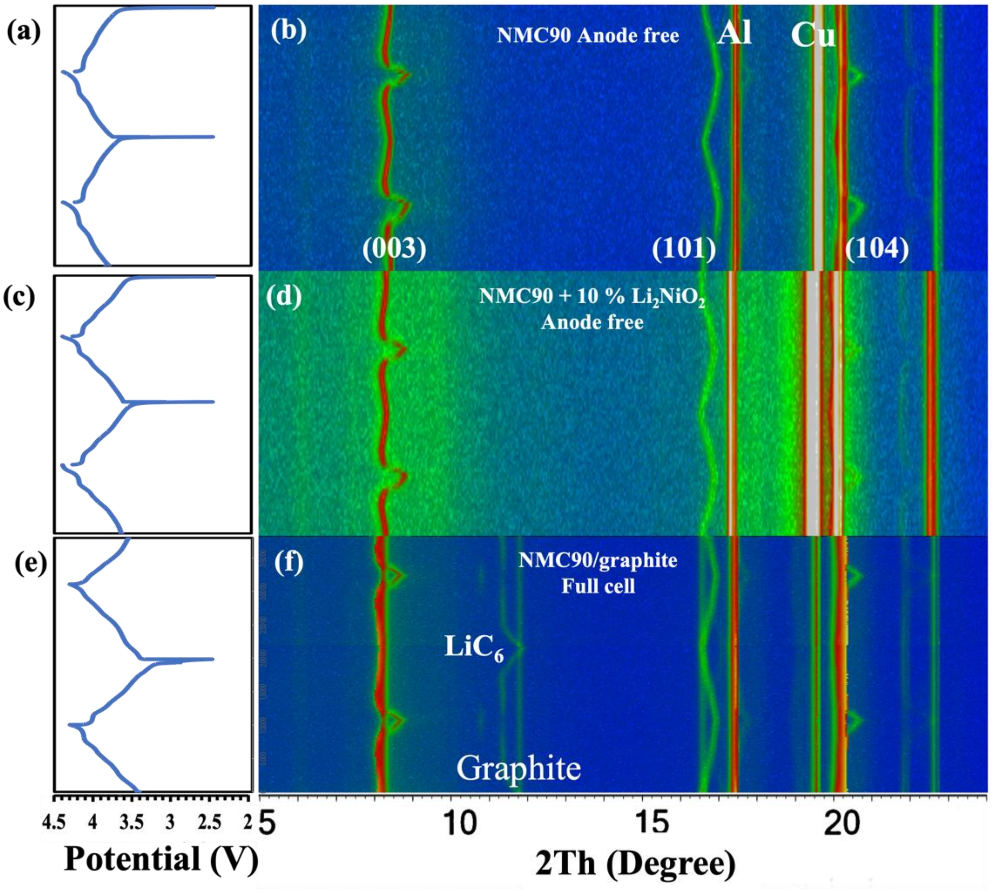

Fig. S16. *Operando* XRD investigation of NMC90 anode-free and graphite pouch cells: Voltage profile and 3D plot of XRD scan. (a, b) NMC90 anode-free, (c, d) NMC90 + 10% Li_2NiO_2 anode-free, and (e, f) NMC90/graphite full cell configurations.

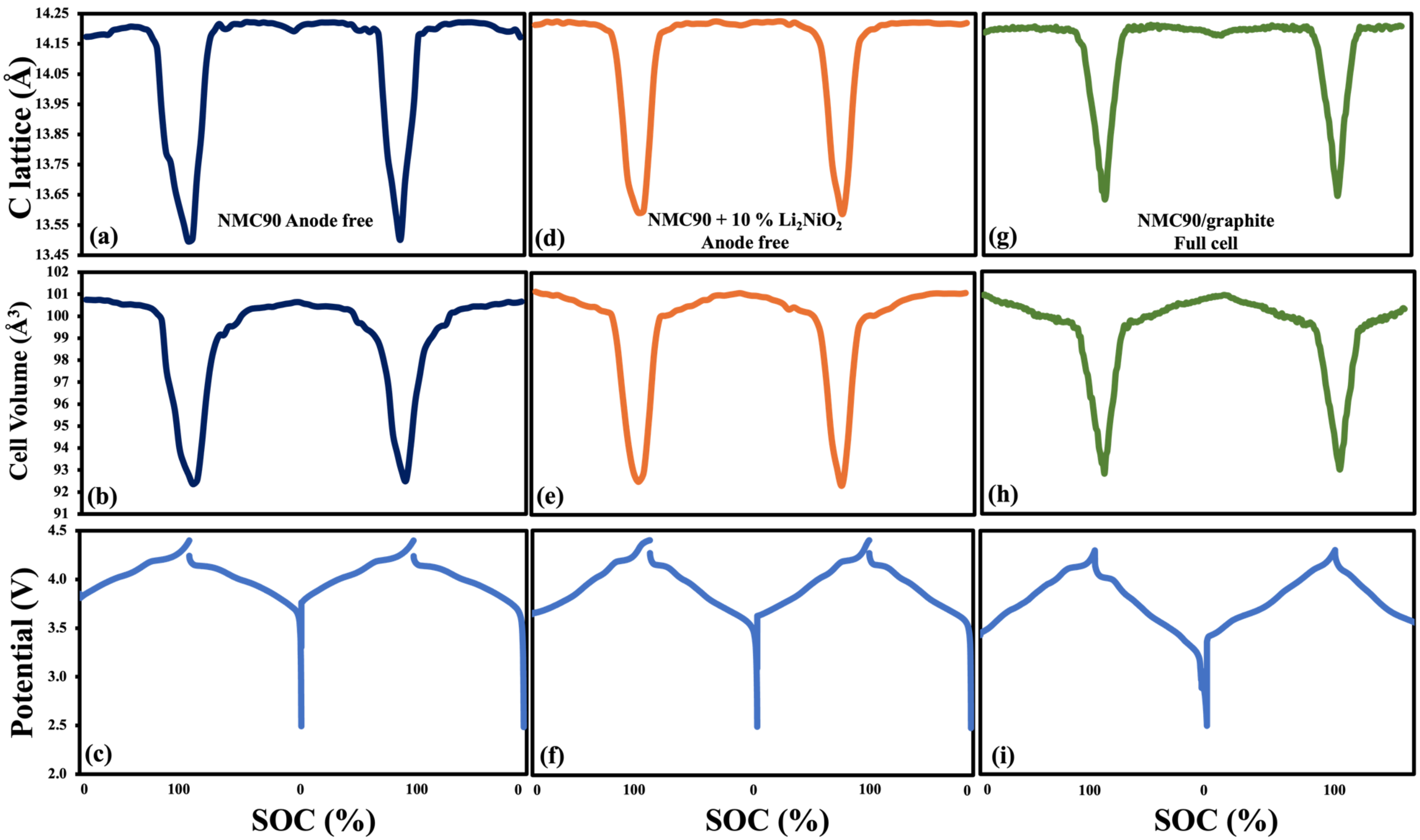

Fig. S17. Rietveld refinement results for *operando* XRD: C lattice unit, cell volume and voltage profile. (a-c) NMC90 anode-free, (d-f) NMC90 + 10% Li_2NiO_2 anode-free, and (g-i) NMC90/graphite full cell configurations.

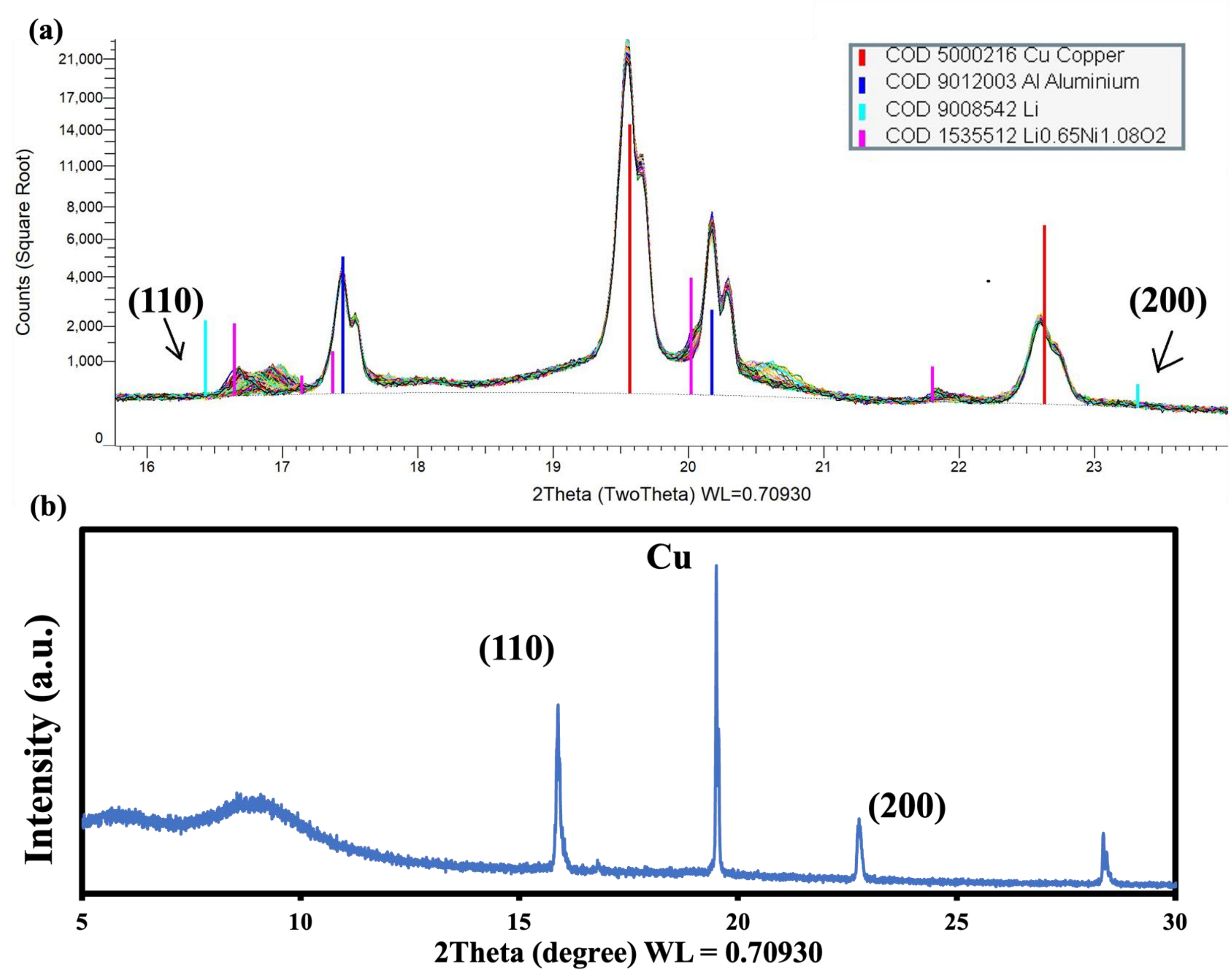

Fig. S18. Fitting for Li species of XRD scans: (a) *Operando* scan of anode-free pouch cell and (b) Control commercial Li metal on Cu foil

5. Supplementary VDO

VDO S1. Anode free NMC90 + 10 % Li_2NiO_2 winding

https://www.dropbox.com/scl/fi/l0yv2euftyh5iqfjemka4/VDO-S1.mp4?rlkey=nxyikiskp25xt368s3jtelv75&dl=0
